# When the brain says “No!”: An MRI study on the neural correlates of resistance to immoral orders

**DOI:** 10.1162/imag_a_00392

**Published:** 2024-12-19

**Authors:** L. Tricoche, A. Rovai, Emilie Caspar

**Affiliations:** Moral & Social Brain Lab, Department of Experimental Psychology, Ghent University, Ghent, Belgium; Translational Neuroanatomy and Neuroimaging Lab, Université Libre de Bruxelles, Brussels, Belgium

**Keywords:** prosocial disobedience, sense of agency, empathy, guilt, cognitive conflict, moral judgement

## Abstract

Milgram’s studies explored psychological and contextual factors influencing (dis)obedience to immoral orders, but the mechanisms preventing individuals from being coerced into causing pain to others remained largely unknown. Our fMRI study investigated the neural correlates of disobedience to such orders, focusing on three phases of the decision-making process: order processing (predecision), action (decision), and outcome and effect processing (postdecision). Within these phases we targeted three sociocognitive (cognitive conflict, sense of agency—SoA, and theory of mind—ToM) and two socioaffective (empathy and guilt) processes. Our findings revealed that participants who engaged the angular gyrus and temporoparietal junction, particularly in the left hemisphere, as well as median prefrontal areas before obeying the command to send a shock—possibly to mitigate cognitive conflict between self and other and to enhance their SoA—were more likely to disobey the experimenter’s instructions to administer a shock to a victim. Additionally, we found involvement of social brain regions during the postdecision phase (encompassing ToM, empathy, and guilt areas), especially in response to shock events, to process the victim’s pain. Higher activity in these regions when obeying orders was associated with a higher rate of prosocial disobedience. This study sheds light on the mechanisms that lead individuals to resist immoral actions under authoritative pressure in an experimental context.

## 1 Introduction

Several historical events have been cited as examples illustrating that obedience to authority can strongly influence human behaviors (Browning, 1992;Zinn, 1997). For instance, qualitative interviews with genocide perpetrators in Cambodia (Caspar, 2024;Hinton, 1998) and Rwanda (Anderson, 2017;Caspar, 2024;Straus & Lyons, 2006), as well as historical accounts from the Nuremberg Trials (Arendt, 1963), demonstrate that obedience to authority is often cited as a justification for participation. Studying people’s ability to question and resist orders, particularly immoral ones, thus appears to be a fundamental aspect of individual autonomy and the functioning of democratic societies. However, experimental research on neurocognitive processes that prevent an individual from being coerced into causing pain to others is very scarce. Previous experimental research has shown that individual characteristics, such as personality traits, explain our susceptibility to others’ influence and our inclination to obey or disobey (Bègue et al., 2015;Milgram, 1974;Passini & Morselli, 2010). However, these studies do not provide evidence of how individuals can switch between obeying an order given by an authority figure and resisting this order.

Quite recently, a number of studies have delved into the impact of obeying orders on various neurocognitive processes related to different phases of decision making, as opposed to acting freely. Using several imaging methods, these studies have revealed that obeying orders to send or not a mildly painful shock to another person compared with acting freely led to decreased activity in brain regions associated with empathy for the victim’s pain (i.e., insula, inferior frontal gyrus, inferior parietal lobule) and guilt (i.e., anterior cingulate cortex, temporoparietal junction, putamen, and caudate) (Caspar, Ioumpa, et al., 2020;Pech & Caspar, 2023). Other studies have extended these findings by demonstrating that following orders, as opposed to acting freely, induces a reduced implicit sense of agency and a diminished explicit feeling of responsibility. This effect may be attributed to a potential reduction in the linkage between decision making, action, and outcome processes (Akyuz et al., 2022;Barlas, 2019;Caspar et al., 2016,^2021^;Caspar, Lo Bue, et al., 2020). Additionally, an EEG study has shown a lower cognitive conflict—indexed by reduced midfrontal theta activity (Cohen & Cavanagh, 2011;Cohen & Donner, 2013)—preceding the decision to administer a painful shock or not, in coerced compared with free-choice decisions (Caspar & Pech, 2023). However, these studies did not investigate the neural mechanisms associated with resistance to orders, as participants merely adhered to the orders given by the experimenter.

To date, only two studies have tried to unravel the neural mechanisms linked to resistance to orders in a moral context. In a virtual replication of Milgram’s experiment, a team of researchers observed that participants exhibited a quicker tendency to harm an avatar following an order when the activity in the right temporoparietal junction (TPJ)—brain region associated with the ability to understand the states of mind of others (Frith & Frith, 2006;Preckel et al., 2018;Saxe, 2010;van Veluw & Chance, 2014)—was reduced with cathodal stimulation compared with sham stimulation (Cheng et al., 2022). This suggests that impairing our capacity to take the perspective of others could reduce reluctance to cause harm under orders. In another study involving the first generation of Rwandese born after the Genocide Against Tutsis, individuals who attenuated their neural response to the pain of others and their feeling of responsibility when obeying orders were more likely to disobey by resisting orders to inflict physical harm (i.e., a mildly painful electric shock) on another participant (Caspar, Gishoma, et al., 2022). The study also found that a higher midfrontal theta activity over frontal areas associated with processing the auditory instruction, suggesting more attentional resources toward the experimenter (Paus et al., 1997;Villena-González et al., 2018), was associated with less prosocial disobedience (Caspar, Gishoma, et al., 2022). Finally, the more the midfrontal theta activity before obeying to send the harmful stimulation, reflecting a higher cognitive conflict (Cohen & Cavanagh, 2011), the more frequently the individual disobeys (Caspar, Gishoma, et al., 2022).

Based on these recent studies, it appears that several neurocognitive processes may facilitate the brain’s ability to resist immoral orders. However, the differences in the populations tested (i.e., participants born to parents who experienced a genocide*vs.*students in a Chinese university), the targeted neurocognitive processes, and the experimental paradigms (i.e., virtual scenarios vs. real electric shocks) challenge the generalizability of these findings. Consequently, the present study aims to employ*f*MRI to investigate how the brain transitions between obeying and resisting immoral orders. We intend to investigate several neurocognitive processes associated with the different phases surrounding decision making, drawing from current research, to examine how these processes may interact with and relate to resistance to immoral orders. As noted byGert and Gert (2008)in the*Stanford Encyclopedia of Philosophy*, definitions of morality can vary widely. In the present study, the term “immoral order” referred exclusively to instructions within the experimental setup that involve inflicting pain on another person in exchange for a monetary reward.

Building upon the aforementioned literature (Akyuz et al., 2022;Barlas, 2019;Caspar et al., 2016,^2021^;Caspar, Gishoma, et al., 2022;Caspar, Ioumpa, et al., 2020;Caspar, Lo Bue, et al., 2020;Caspar & Pech, 2023), our focus in this study centers on two socioaffective processes, namely empathy and guilt, and three sociocognitive processes, including the sense of agency, cognitive conflict, and mentalizing—all of which are linked to different phases of decision making, from predecision to postdecision. Empathy refers to the ability to share the affective and emotional state of another person (Lamm et al., 2017). Specifically, empathy for pain engages two key regions: anterior insula (AI) and anterior median cingulate cortex (aMCC) (Preckel et al., 2018), while also recruiting the inferior frontal gyrus (IFG), the supramarginal gyrus (SMG), and somatosensory areas (Maliske et al., 2023). Guilt, on the other hand, occurs when we cause pain to others, leading to a desire for reparation (Ortiz Baron et al., 2018). The neural correlates of guilt are often associated with activity in anterior cingulate cortex (ACC) and TPJ, although recent research has identified the putamen, the precuneus, and the SMG as additional guilt-related brain regions (Bastin et al., 2016,^2021^). Both empathy and guilt are recognized as crucial processes linked to prosocial decision making (Bastian et al., 2011;Eisenberg, 2000;Hein et al., 2010;Maliske et al., 2023;Preckel et al., 2018), and are susceptible to alteration in situations of obedience (Caspar, Ioumpa, et al., 2020,^2022^). As these two socioaffective processes occur when witnessing painful outcomes inflicted on someone else (Decety, 2011;Lamm et al., 2017), they should be involved during the postdecision phase, that is, when a mildly painful electric shock is sent to the victim.

Sense of agency (SoA) and cognitive conflict are two sociocognitive processes. SoA refers to our ability to make voluntary actions and our feeling to be at the origin of these actions and their associated consequences (Gallagher, 2000). The literature indicates that the SoA can be measured using implicit methods, based on time perception (Haggard, 2017;Moore & Obhi, 2012). It has been shown that performing voluntary actions leads to a compression of time between the action and its outcome compared with performing the same actions involuntarily, an effect referred to as temporal binding (Haggard et al., 2002;Noel et al., 2023). Previous MRI research has suggested that temporal binding, especially for neutral outcomes, involves the supplementary motor area (SMA) (Farrer & Frith, 2002;Kuhn et al., 2012). However, in contexts with social significance, it has been associated with the middle frontal gyrus (MFG) (Caspar et al., 2021). Given that SoA has been found to be reduced under coercion, this may suggest that participants perceive themselves as not being the initiators of these actions and disengage from the resulting consequences (Caspar et al., 2016;Schwarz et al., 2023;Villa et al., 2022). This aligns partially with the agentic state theory developed byMilgram (1974)and the widely spread “just following orders” defense postwars, where individuals perceived themselves as passive and not responsible executors because they were not at the origin of the decision (Anderson, 2017;Caspar, 2024;Hinton, 1998). Against this background, SoA occurs between the decision-making phase and the outcome phase, since the measurements based on time perception occur between an action and its resulting consequence. Cognitive conflict arises when incongruent or competing information occurs, requiring specific resources and processing to resolve the discrepancy between the information received and the decision making (Cohen, 2014;Ridderinkhof et al., 2004). It involves the ACC and the medial frontal cortex (MFC) (Cohen, 2014). Choosing between following the experimenter’s order or following one’s own judgment by disobeying could lead to a conflict during the predecision phase, solved by weighing the pros and cons (i.e., gaining more money and being impacted by the “authority bias effect” or being moral and empathizing with the victim) (Götz et al., 2023;Mellers et al., 1998;Shafir et al., 1993), which leads to a specific action in the decision-making phase. In that case, internal motivations are expected to be at the root of the cognitive conflict (Amodio et al., 2008), grounded in the belief that agents are inherently prosocial and moral. Mentalizing abilities refers to our capacity to infer others’ states-of-mind, involving the Theory of Mind network (ToM), mostly composed of the TPJ extending to the posterior part of the superior temporal sulcus (pSTS), the precuneus extending to posterior cingulate cortex (Prec/PCC), and the dorso- and ventromedian prefrontal cortex (dmPFC, vmPFC) (for reviews see for example (Amodio & Frith, 2006;Preckel et al., 2018). In line with Cheng and collaborators’ tDCS study (Cheng et al., 2022), the ToM network is also expected to be involved in prosocial disobedience, as mental state information is deemed necessary during moral decision making and prosocial behaviors, such as deciding whether to obey or cause pain to someone else in the present study (Maliske et al., 2023;Ye et al., 2015;Young & Tsoi, 2013).

We used a recently developed paradigm designed to generate resistance to orders to inflict harm on another person (Caspar, 2021) and adapted it for use in*f*MRI. We recruited a total of 57 young adults, being in the role of an agent receiving direct orders from an experimenter while being scanned. On each trial, they were ordered by the experimenter to send or to not send a mildly painful electric shock on a victim’s hand visible on a screen. Following the order, agents had to decide whether to send a shock or not (i.e., obey or disobey orders) by pressing one of two keys. To question the morality of the agent’s action, pain caused to the victim was associated with a financial gain for the agent. If agents chose to send a shock, they indeed earned +€0.05, but they did not receive the reward if they chose not to send the shock. This experimental design allowed us to study the differences between following an order and refusing to follow it, though within a very specific context. Thus, the disobedience induced in agents can only be considered and interpreted within this particular experimental framework, in which agents faced bimodal decisions (whether to administer a shock or not) and were tested under a controlled protocol approved by an ethics committee, which explained that while the shocks were painful, they posed no actual danger to the victim.

The selected time windows for targeting our processes of interest were defined to investigate the entire trial sequence with different steps in the decision-making process, encompassing processing, decision, action, outcome, and posteffects (Caspar et al., 2021). Using frequentist and Bayes factors, we computed different MRI contrasts to target the epochs of our processes of interest. Notably, we contrasted obedience trials together, when people received an order to send a shock to the victim or not. We then analyzed whether specific processes involved when people obey orders further correlate with the number of times they choose to disobey. We also focused specifically on trials involving immoral orders, that is, when the experimenter gives the orders to send a shock, and we contrasted trials where participants obeyed that order (i.e., obedience trials) or disobeyed that order (i.e., prosocial disobedience trials). Then, we used a contrast to isolate viewing or not the victim’s pain. Following the act of sending a shock, we expected to observe increased activity in regions previously found associated to pain- and guilt-related brain networks, as well as the ToM network. This heightened activity, in turn, was anticipated to lead to a greater likelihood of disobedience in subsequent trials. Specifically, we predicted that disobedience would occur only if individuals could maintain sufficient neural activity linked to the victim’s pain during the postdecision phase. Supporting this prediction, a previous EEG study showed that an attenuated neural response to a victim’s pain was linked to a lower rate of prosocial disobedience (Caspar, Gishoma, et al., 2022). Furthermore, individuals with reduced processing of auditory instructions from the experimenter (as measured in frontal areas) were shown to disobey more frequently in a previous study (Caspar, Gishoma, et al., 2022). Therefore, we hypothesized that only individuals enhancing brain activity after receiving the instruction and until their decision making, potentially associated with cognitive conflict with the experimenter’s order or with maintaining their SoA, would engage in disobedience. Particularly, we expected that agents with more cognitive conflict (higher decision time) and higher SoA (higher temporal binding) could resist immoral orders more, with associated modulations in the related areas (SMA, MFG, ACC, MFC) during the predecision and decision-making phases.

## 2 Methods

### 2.1 Participants

The study and its hypothesis were preregistered on OSF (https://osf.io/n8ce5). An a priori power analysis indicated that a sample size of 36 was required for ANOVA to detect a medium effect size ($\eta_{p}$² = 0.06) on the within-subject Instruction x Choice interaction and a sample size of 38 for correlation analyses (*r*= 0.5) between ROI activity and %Pro_disob, Interval Estimates (IE) scores, or pain score, with a power of 95% and a Type I error of 5%. We increased the sample size to account for potential data loss. Participants were recruited via social media. Fifty-seven young adults (33 who identified as females and 24 who identified as males, range: 19–36 years, mean: 23.7 ± 4.2 years) completed the*f*MRI session and played the “agent” role. They were overall French, Dutch, or English speakers, with a normal or corrected vision, without any MRI contraindication, and without neurological or psychological known disorder. One participant inverted the mapping associations (shock and no shock) during the Agency run and was removed from the analyses for this run. Another participant was removed from analyses in the Empathy run due to a malfunction of the audio headset during this session. A third participant was completely excluded from the fMRI analyses due to a technical problem during the MRI data acquisition. We conducted a linear trend analysis with contrasts -1 0 1 to ensure that participants correctly reported shorter IE for short delays and longer IE for longer action–outcomes delays. No participants were excluded based on the linear trend analysis for the three delays used. The final samples thus consisted of 56 participants for fMRI analyses and 56 participants in each run for behavioral analyses (excluded participants were not the same). All participants signed an informed consent and received a financial compensation between €30 and €40. The study was approved by the local ethics committee of Erasme Hospital (Belgium, reference: SRB2022127).

### 2.2 Procedure

Participants came to the MRI scanner alone and were introduced to an unknown age- and gender-matched partner, who acted as a second participant. The participant took the role of “agent,” whereas the partner took the role of “victim.” To make the real participants believe that the other person was also a participant, the experimenter welcomed them at the same time in the waiting room of the hospital. Additionally, participants were told that two different advertisements had been sent for the role of agent and for the role of victim, and thus their roles were already decided based on where they sent their application. In addition, all the information was provided similarly to the two participants, and the confederate asked questions such as any naïve participants would do.

The different neurocognitive processes were targeted in two separate MRI runs, both based on the same experimental paradigm. This decision was informed by a previous study indicating that if the consequence of an action occurs more than 4 seconds after the action, modulations of the SoA with the temporal binding method no longer lead to measurable changes in time perception (Buehner & Humphreys, 2009). To distinguish brain activity related to motor response from that related to processing the pain of the victim, relatively long jittered action–outcome delays had to be used, precluding the use of time estimates. In the Agency run (targeting the predecision phase, the decision-making phase, and the postdecision phase for posteffects), agents completed a temporal binding task, whereas in the Empathy run (targeting the postdecision phase for outcome and posteffects), agents rated a pain scale.

**Fig. 1.**
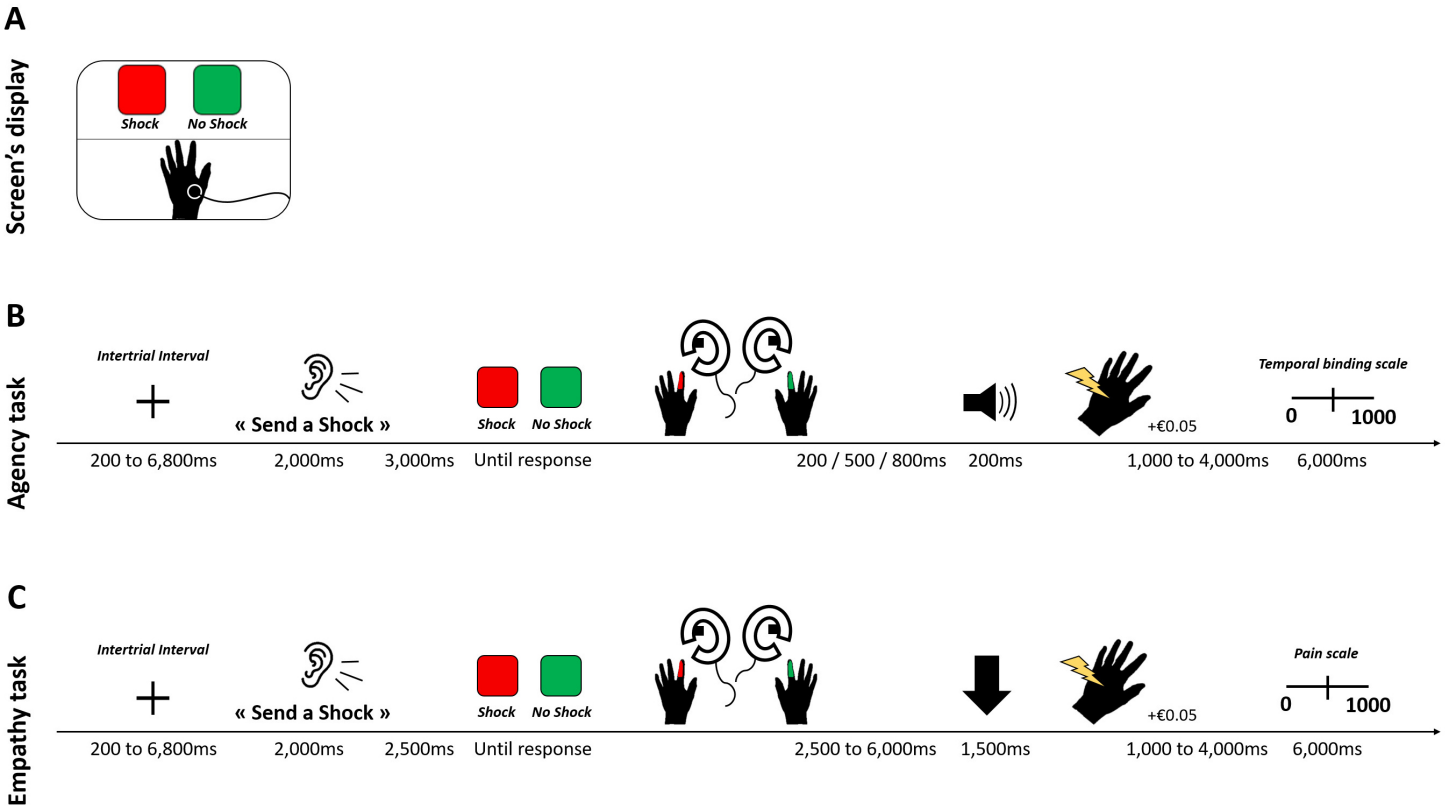
The screen seen by agents in the MRI scanner was split into two sections: the upper displayed the experimental interface, and the lower showed a live video feed of the victim’s hand (A). Trial sequence of the Agency (B) and Empathy (C) runs. After receiving the auditory instruction (“Send a shock,” “Do not send a shock”), agents must decide to obey or not to the experimenter order by pressing one of the two keys. Then, a tone occurred (for Agency) or an arrow was displayed (for Empathy). According to the outcome on the victim’s hand (received shock or not), the agent could earn + €0.05. At the end of the trial, agents used the temporal binding scale (Agency) and the pain scale (Empathy).

After signing the consent forms, participants in the role of the agent were trained for the two runs (Agency and Empathy) to ensure they understood the tasks and correctly estimated the delays between the keypress and the tone for the Agency run (see below). No participants required more than 12 trials to successfully complete the training. Then, the pain threshold was determined for both participants in front of each other. We placed two electrodes on their left hand, which were connected to a Digitimer DS7A sending electric stimulations setup to be at the pain threshold. The electrodes were placed on the abductor pollicis muscle as it produced a visible muscle twitch, visible when a shock is delivered. Visualizing a painful stimulation is indeed important to trigger an empathic reaction in the brain of the observer (Keysers & Gazzola, 2014;Krishnan et al., 2016). The experimenter increased the threshold by steps of 2 mA until determining a threshold that was mildly painful for both participants. Participants were instructed to indicate when the sensation of the administered shock changed from unpleasant to painful. To confirm their certainty, the experimenter reiterated the shock at the same intensity, seeking confirmation from the participants. Once they affirmed that the discomfort had indeed escalated to pain, the designated pain threshold was established. This procedure ensured that our participants knew that the shocks were real and how painful they were. Unknown to the participants, during the acquisition, the threshold of the victim was reduced up to a threshold that was not painful, but still triggered a visible muscle twitch. After the pain threshold procedure, the confederate playing the role of the victim sat at a table located outside the MRI scanner, connected to the electric device, and the participant playing the role of the agent was placed in the scanner with a joystick in each hand. In front of them was a screen split into two sections (Fig. 1A): the upper displayed the experimental interface and the lower showed a live video feed of the victim’s hand, ensuring that the agent could observe the hand throughout the task and confirming that the video was not prerecorded. In the MRI scanner, participants completed a second training session (12 trials) only for the Agency run, to help them get used to estimating time intervals with the MRI scanner’s cyclic noise.

There were two different MRI runs: one for Agency and one for Empathy, using the same experimental protocol. For both runs, on each trial, the agent was asked by the experimenter to send or not a real mildly painful electric shock to the victim (“Send a shock”, “Do not send a shock”). The instructions were actually prerecorded to control precisely the timing of the audio instructions. To make participants believe that the instructions were really given in real time by the experimenter, there were six prerecorded instructions for each order, with small variations in the voice of the experimenter, similarly toCaspar, Ioumpa, et al. (2020). After having received the instruction, agents had to decide to obey or not the experimenter’s orders by pressing the corresponding “shock” or “no shock” button. To indicate which button was associated with shock and no shock on each trial, an image appeared, with a red square labeled “shock” and a green square labeled “no shock,” displayed on the left and right parts of the screen. The mapping of the two buttons varied randomly to avoid participant’s response anticipation. Each time agents decided to give a shock, they earned + €0.05 in addition to their basic remuneration. For each run, the experimenter ordered agents to “send a shock” in 80 trials and to “not send a shock” in 40 trials. The sequence of orders given by the experimenter to shock or not the victim was random and differed between agents. Each MRI run lasted 20 minutes. In total, agents performed 240 trials, 120 in each MRI run.

In pilot studies, participants never disobeyed any orders, despite previous studies conducted with EEG showing a high prosocial disobedience rate (Caspar, Gishoma, et al., 2022). Those pilot participants later reported that they did not dare to disobey in an experiment taking place in a hospital. This is consistent with Milgram’s studies showing that obedience decreases when his study was conducted in a less credible location than in Yale University (Milgram, 1974). Without participants disobeying orders, we could not study disobedience. We thus offered participants the possibility of not following orders by explicitly mentioning this option in the instructions: “I will ask you on each trial whether to send a shock to the victim or not. You will then choose to press one or the other button. Please know that you are not obliged to follow my instructions, I cannot force you to do anything, ultimately it is your decision.”

### 2.3 Task and trial sequence

Agents completed two tasks in distinct counterbalanced, Agency and Empathy, runs. During the Agency run, at each trial, agents decided to obey or not to the experimenter order by pressing one of the two keys. Then, a 400 Hz-tone followed the keypress after a 200, 500, or 800 ms pseudorandomized delay. When a shock was given to the victim, it was delivered simultaneously with the tone occurrence. Agents were asked to estimate the action–outcome delay corresponding to the interval between the keypress and the tone (Caspar et al., 2016). A temporal binding scale from 0 to 1,000 ms was displayed on the screen. Agents had 6 seconds to estimate the interval by adjusting a red position marker on the scale, using the joysticks. Trial sequence of the run is illustrated inFigure 1B.

During the Empathy run, after agents decided to obey or not the experimenter order by pressing one of the two keys, an arrow pointing to the video of the victim’s hand was displayed on the screen during 1,500 ms. This ensured that agents focused on the hand during the outcome (shock/no shock). The shock was delivered between 2.5 and 6 seconds after the keypress to ensure no overlap between the motor response of the keypress and the processing of the victim’s pain (Caspar, Ioumpa, et al., 2022). Then, a pain rating scale appeared randomly in 20% of the trials (24/120 trials). It was ranged from 0 (“not painful at all”) to 1,000 (“very painful”). Agents had 6 seconds to rate the intensity of the victim’s pain, associated with the received shock they saw, by adjusting a red position marker on the scale, using the joysticks. If no shocks were delivered on that trial, agents were asked to report that the shock was “not painful at all.” Trial sequence of the run is illustrated inFigure 1C.

### 2.4 Questionnaires

Agents completed several questionnaires before and after the MRI session and answered debriefing questions to investigate several individual and social dimensions, and their potential modulation on the results. One or two weeks before the experiment, at home, participants completed several questionnaires and sent them back by mail. They completed the Aggression-Submission-Conventionalism (ASC) scale (Dunwoody & Funke, 2016) to investigate the relationship with authority, referring to the idea that some people need very little situational pressure to submit to an authority figure and to accept illegitimate orders to hurt others, while other individuals need significantly more. The Attitude for Money (AfM) scale (Yamauchi & Templer, 1982) was completed to investigate whether the impact of money reward obtained after each shock could be different according to the standard money attitude of each participant. To investigate the difference of sensitivity to distinct competing issues of morality, participants completed the Moral Foundation (MF) questionnaire (Graham et al., 2011). The Resistance to Peer Influence (RPI) scale (Steinberg & Monahan, 2007) was used to investigate how much people think that another person can influence them. We also asked participants to complete the Short Dark Triad (SD3) scale (Jones & Paulhus, 2014), investigating three socially aversive traits: Machiavellianism, narcissism, and psychopathy. Finally, thoughts and feelings of empathy were subjectively assessed with the Interpersonal Reactivity Index (IRI) (Davis, 1980). Just after the MRI session ending, agents completed an additional questionnaire: The Social Identification with the Experimenter (SocId) questionnaire (Steffens et al., 2014). It investigates whether agents who identified the most themselves to the experimenter were also more prone to follow the orders during the experiment. Participants finally answered debriefing questions that can be found inAppendix A. These questions investigated (1) whether participants voluntarily decided to disobey, (2) different feelings among which the explicit feeling of responsibility by rating scales from “not at all” to “extremely” (i.e., How responsible did you feel when you were agent?), (3) why agents obeyed or disobeyed orders, also by using scales from “not at all” to “extremely” (i.e.,*because there were too many shocks, for moral reasons, to win more money, because it was the aim of the experiment*…), (4) the estimated number of trials where agents disobeyed (prosocially or antisocially), and (5) how agents felt during the experiment by open-ended question.

### 2.5 Behavioral data and analyses

Analyses were conducted using R (RStudio, v.4.0.0). For both runs, we measured the Prosocial Disobedience rate (%Pro_disob), corresponding to the number of times the agents refused to deliver a shock to the victim, despite the “Send a shock” order given by the experimenter.

Implicit SoA was targeted by measuring the temporal binding score, similarly to previous studies (Caspar et al., 2016;Haggard, 2017;Moore & Obhi, 2012). During the Agency run, participants were asked to estimate the duration (0 to 1,000 ms) between their keypress (action) and the tone associated with the shock/no shock (outcome). These interval estimates (IE), referring to the temporal binding score, were then transformed into z-scores to reduce intersubject variability and personal strategies when using the scale (i.e., some participants prefer using the high part of the scale, while others the low part): z-score =$\frac{IE_{i}-meanIEs}{sdIEs}$, where*i*corresponds to one trial. We also calculated the mean difference between the IE and the actual delay (error-score), as a second indicator of temporal binding. Then, higher IEs (i.e., positive z-score or positive error-score) are associated with a lower SoA (less feeling to be responsible of our own actions), and reciprocally. We used both z-score and error-score because both were used in previous studies, making it difficult to directly compare the results (Caspar et al., 2021;Caspar, Gishoma, et al., 2022;Dewey, 2024).

Empathy was estimated with the pain scale (values between 0 to 1,000) used by agents to report the intensity of the pain caused on the victim according to the received/not received shock. The higher the estimated intensity on the scale, the higher the empathy for victim’s pain. For analyses we used the mean pain scores.

Finally, for both runs, Decision Times (DTs, in ms) were calculated, and corresponded to the delay between the screen display of the Shock/No Shock key associated responses (i.e., left key = Shock, right key = No Shock, or reversely) and the keypress of the agent.

For each run separately, two-way Instruction (“Send a shock”, “Do not send a Shock”) X Choice (Obedience, Disobedience) ANOVAs were conducted on the temporal binding scores, the empathy score, and the DT, where Instruction and Choice were within-subject factors. Pairwise comparisons with False Discovery Rate (FDR) correction for multiple comparisons were conducted to interpret significant interactions (Benjamini & Hochberg, 1995). Effect sizes ($\eta_{p}$²) and confidence intervals at 95% ($CI_{95})$are also reported. We also conducted ANOVAs on the %Pro_disob controlling for the effects of Run (Agency, Empathy) and Order (completing the Agency run or the Empathy run first).

Using Pearson correlation tests (or the equivalent nonparametric Spearman test when the normality criterion was not complied), we conducted exploratory analyses with correlations between the %Pro_disob and the temporal binding z-score or error-score, as well as the empathy score (using both the mean and the medium pain values), and DT. The %Pro_disob, but also the antisocial disobedience rate, was correlated with the subscales assessing their subjective experiences and the reasons of disobedience subscales completed during the debriefing phase.

Linear regressions were conducted as an exploratory analysis between the %Pro_disob and the questionnaires’ scores (ASC, AfM, MF, RPI, SD3, IRI). The procedure is detailed inAppendix B. Significance level was set at*p*< 0.05 for all analyses.

To complement these frequentist statistics, we also conducted Bayesian analyses using JASP. For ANOVAs, we calculated the Bayes Factor inclusion ($BF_{incl}$) that compares all models including an effect (i.e., Instruction) on all models that do not include the effect. For correlation tests, we calculated the Bayes Factor 10 ($BF_{10}$). We used the standard references where an extreme, strong, moderate, or anecdotal evidence for the null hypothesis (H0) was set for a$BF_{incl}$or$BF_{10}$<0.001, <0.01, <0.33, <1, respectively, whereas extreme, strong, moderate, or anecdotal evidence for the alternative hypothesis H1 was set for a BF >100, >10, >3, and >1 (Keysers et al., 2020;Wagenmakers et al., 2018).

### 2.6 fMRI data acquisition

MRI images were recorded using a 3.0-Tesla Siemens trio scanner and a 32-channel head coil at Erasme Hospital in Brussels (Belgium). Two runs, one for Agency and one for Empathy, of functional images were recorded in a single-shot echo planar imaging (EPI) sequence with the following parameters: matrix = 96 x 96; number of slices = 45; TR = 2.2 seconds; TE = 20 ms; flip angle = 90°; voxel size = 3 x 3 x 3 mm³; slice thickness = 3 mm. Between the two functional runs, T1-weighted 3D structural images were acquired (matrix = 240 x 192; TR = 8.2 ms; TE = 3.1 ms; flip angle = 12°; voxel size = 0.94 x 0.94 x 0.5 mm³, slice thickness = 1 mm). A functional resting-state acquisition was added at the end of the experiment, but it was not analyzed in the present study.

### 2.7 fMRI data preprocessing and processing

MRI data were preprocessed using fMRIPrep 20.2.0 (Esteban et al., 2019). T1 images were segmented and normalized to the MNI space. Functional images were realigned, slice-time corrected, coregistered, and warped to the normalized anatomical image (for a full report of the preprocessing pipeline seehttps://osf.io/xcgak/). Using SPM12, images were spatially smoothed with a 6 x 6 x 6 mm³ kernel.

At the first level, we defined separate regressors for “Send a shock” and “Do not send a shock” instructions and for Obedience and Disobedience choices, with Agency and Empathy trials modeled in separated runs. For the Agency run, activation was modeled as epochs with three distinct onset times. The first one started with the screen display of the Shock/No Shock key associated responses and lasted until the tone, in order to target the decision-making phase for action. In fact, this epoch corresponded to the period between the decision making and the outcome, including the motor action. The second one was the 3,000 ms delay after the auditory instruction, in order to target the predecision phase for auditory processing. Finally, the delay between the tone and the interval estimate scale display was used to target the postdecision phase for posteffects, as it was the period after the outcome (Zhu et al., 2019). As this delay was the same for the two runs, the posteffect period was investigated for both Agency and Empathy runs (i.e., between the arrow display and the pain scale display), and merged together in the analyses (controlling for the Run effect).

For the Empathy run, we also modeled the hemodynamic response using the onset time starting with the display of the arrow pointing to the video of the victim’s hand, in order to target the postdecision phase for outcome (Caspar, Ioumpa, et al., 2020).

A regressor of no interest included all the other events: (1) the auditory instructions, (2) the visual display of the temporal binding scale, (3) the visual display of the pain scales, and (4) the keypress and the tone (excepted for SoA in Agency run). The six motion regressors were also added in the model.

### 2.8 fMRI data analysis

MRI data analyses were performed using SPM12.

#### 2.8.1 Whole-brain analyses

At first level, we defined different contrasts for each of the GLM models (predecision, decision making, and postdecision for outcome or posteffects) (seeTable 1). These contrasts allowed us to target specifically some of the neurocognitive processes of interest. The first contrast of interest was the [“Send a shock”/Disobedience > “Send a shock”/Obedience] and its reverse contrast to investigate the neural signature of prosocial disobedience. In this contrast, the order received was similar (i.e., “give a shock”), but the agent’s action differed (i.e., obey or disobey). For this contrast, we analyzed the epochs linked to the pre- and decision-making phases. Analyses on the epochs associated with the postdecision phase would be unreliable in this contrast, as they would contrast a trial with no shocks (i.e., when agents hear “Send a shock” but disobeyed) to a trial with a shock (i.e., when agents hear “Send a shock” and obey, thus actually delivering a shock). In a second contrast, we isolated the brain processes involved when obeying orders, and we contrasted [“Send a shock”/Obedience > “Do not send a shock”/Obedience] and its reverse contrast to investigate obedience trials, depending on the orders received. In this contrast, we analyzed the epochs associated with all our phases of interest. To isolate the effect of seeing the victim’s pain, we defined the contrast [“Send a shock”/Obedience + “Do not send a shock”/Disobedience] > [“Do not send a shock”/Obedience + “Send a shock”/Disobedience]. For this contrast, we focused on decision-making and postdecision phases. For the decision-making phase, agents do not already see the victim receiving or not a shock. But when agents make their decision and press the key, they already know precisely what the outcome of their actions will be, as action–outcomes were always entirely predictable. We thus also decided to include this phase in this contrast. The results obtained for the other contrasts: [“Do not send a shock”/Disobedience > “Send a shock”/Obedience] and [“Send a shock”/Disobedience > “Do not send a shock”/Obedience] are described inAppendix C. Finally, we also defined a global [“Send a shock” > “Do not send a shock”] contrast to investigate the effect of the received instruction. For this final contrast, we focused on the predecision phase, which corresponds to the moment agents heard the instructions.

**Table 1.** Used contrasts in the analyses.

Contrast (and reverse)	Focus	Targeted phases
[“Send a shock”/Disobedience > “Send a shock”/Obedience]	Focus on the agent’s action, with a similar order received	Predecision (auditory processing) and decision making (action)
[“Send a shock”/Obedience > “Do not send a shock”/Obedience]	Focus on obedience trials, depending on the order received	All phases (auditory processing, action, outcome, and posteffects)
[“Send a shock” > “Do not send a shock”] instruction	Focus on the auditory processing of the experimenter’s orders	Predecision (auditory processing)
[(“Send a shock”/Obedience + “Do not send a shock”/Disobedience)] > [(“Do not send a shock”/Obedience + “Send a shock”/Disobedience)]	Focus on when the victim receives a shock compared with when the victim did not receive a shock	Decision making (action) and postdecision (outcome and posteffects)

A contrast [(“Send a shock”/Disobedience > “Do not send a shock”/Disobedience) > (“Send a shock”/Obedience > “Do not send a shock”/Obedience)] to investigate the agent’s brain differences when the victim received a shock compared with when the victim did not receive a shock was not possible in our experimental design, which depends on participant’s behaviors. Some agents indeed either administered few shocks, or not easily disobeyed or neither/always adopted an antisocial behavior. To ensure the reliability of the*f*MRI results emerging from this contrast, and based on a previous study (Caspar, Ioumpa, et al., 2020), we had to remove agents who did not have a sufficient number of trials (<5 trials) in one or several of the four conditions (i.e., “Send a shock”/Obedience, “Do not send a shock”/Disobedience, “Do not send a shock”/Obedience, “Send a shock”/Disobedience). As too many agents were discarded (42/56, seeSection 3.1), we did not include it in the analyses. This was mostly due to the low antisocial disobedience rate observed (“Send a shock”/Disobedience). Importantly, this exclusion criterion based on number of trials was also applied for the other used contrasts of interest. As this full model could not be involved in the analysis, the first contrast of interest [“Send a shock”/Disobedience > “Send a shock”/Obedience] was also investigated by controlling for the activity found in the contrast comparing obedience to send a shock and obedience to not send a shock. We then applied an exclusive mask using the significant clusters obtained from the [“Send a shock”/Obedience > “Do not send a shock”/Obedience] contrast. Concretely the exclusive mask filtered out all significant voxels identified in the [“Send a shock”/Obedience > “Do not send a shock”/Obedience] contrast. This step ensured that the resulting voxels in the [“Send a shock”/Disobedience > “Send a shock”/Obedience] contrast represented only the brain activity associated with disobedience.

We also conducted two exploratory analyses. In the first one, we compared the obedience to send a shock trial at time*t*that led to prosocial disobedience*versus*obedience to send a shock during the next trial at time*t*+ 1. We hypothesized to find higher brain activity at time*t*during the postdecision phase, when agents disobeyed in the following trial at time*t*+ 1. These exploratory but nonsignificant results are described inAppendix D. In the second one, we investigated whether brain activity at time*t*during the postdecision phase processing outcomes could influence the behavioral decision at time*t*+ 1. The methodology and (nonsignificant) results of this analysis are detailed inAppendix E.

For all our contrasts, we used a significance threshold of*p*< 0.05 (FWE corrected for multiple comparisons) at the cluster level, with an initial voxel-wise probability threshold of*p*< 0.001 uncorrected, with the exception of the analysis using the exclusive mask which was reported with a threshold of*p*< 0.005 uncorrected.

#### 2.8.2 ROI analyses

Based on the significant clusters obtained at whole-brain level, using Marsbar, we built ROIs as the intersection of 10 mm radius spheres centered on the local maximum of each cluster. In each ROI, the activity was averaged across all voxels. When the same region was found in several epochs, we estimated the coordinates to best fit all the clusters. Fifteen ROIs were defined: bilateral anterior insula (AI), bilateral inferior occipital gyrus extending to angular gyrus (IOG/AG), bilateral precentral gyrus (PreG), bilateral supramarginal gyrus (SMG), bilateral superior parietal lobule (SPL), bilateral temporoparietal junction (TPJ), precuneus extending to posterior cingulate cortex (Prec/PCC), and bilateral supplementary motor area (SMA). We also added two prefrontal regions (ventromedian prefrontal cortex extending to anterior cingulate cortex [vmPFC/ACC] and dorsomedian prefrontal cortex [dmPFC]) in this analysis as these two regions are involved in social functions (Amodio & Frith, 2006;Preckel et al., 2018). The used coordinates of the 17 ROIs are reported inTable 2. The mean beta values were extracted from both the [“Send a shock”/Obedience > “Send a shock”/Disobedience] (i.e., same order but different behaviors) and the [“Send a shock”/Obedience > “Do not send a shock”/Obedience] (i.e., different orders but similar behavior) contrasts for each ROI. Spearman correlations were conducted between the mean beta values and the %Pro_disob. An FDR correction for multiple tests was applied, as a correlation coefficient was calculated for the 17 ROIs. Eight correlations tests were conducted, one for each epoch and contrast (i.e., four epochs by two contrasts).

**Table 2.** MNI coordinates (in mm) of each defined ROI used to conduct correlations analyses between the mean beta values and %Pro_disob.

ROI	Coordinates		
	x	y	z
Left AI	-34	18	9
Right AI	41	21	-1
Left IOG/AG	-52	-74	-1
Right IOG/AG	52	-74	-4
Left PreG	-54	0	39
Right PreG	54	7	42
Left SMG	-68	-27	39
Right SMG	68	-27	39
Left SPL	-38	-47	63
Right SPL	38	-47	63
Left TPJ	-61	-43	24
Right TPJ	68	-43	21
Prec/PCC	5	-54	33
Left SMA	-4	16	51
Right SMA	3	21	48
dmPFC	-16	23	60
vmPFC/ACC	3	54	21

The coordinates were obtained based on the significant clusters found for [“Send a shock”/Obedience > “Send a shock”/Disobedience] and [“Send a shock”/Obedience] > [“Do not send a shock”/Obedience] contrasts at whole-brain level.

Both frequentist and Bayesian analyses were conducted, and effect sizes were reported. Correlations between the percentage of obedience to send a shock and the ROIs were also conducted as a supplementary analysis and reported inAppendix Fin order to investigate whether the activity of some brain regions was involved in the antisocial obedience. Finally, we explored the correlations between the beta values of the ROIs (using the [“Send a shock”/Obedience > “Send a shock”/Disobedience] and the [“Send a shock”/Obedience > “Do not send a shock”/Obedience] contrasts) and the IE’s z-score, IE’s error score and pain scores.

#### 2.8.3 Qualitative analysis using NeuroQuery generated maps

We used the NeuroQuery website (https://neuroquery.org/) to investigate our neurocognitive processes of interest (Dockès et al., 2020). We entered keywords in NeuroQuery to generate brain maps based on the NeuroQuery corpus, and to compare them with the maps obtained for each phase (predecision, decision making, and postdecision) in our contrasts of analysis. Based on our hypotheses, the used keywords were (1) cognitive conflict, (2) agency, (3) empathy, (4) guilt, and (5) theory of mind. As empathy differs according to the context, we specified our query by adding the “pain” and “hand” terms in order to fit with our paradigm.

## 3 Results

### 3.1 Behavioral results

#### 3.1.1 Percentage of disobedience

**Fig. 2.**
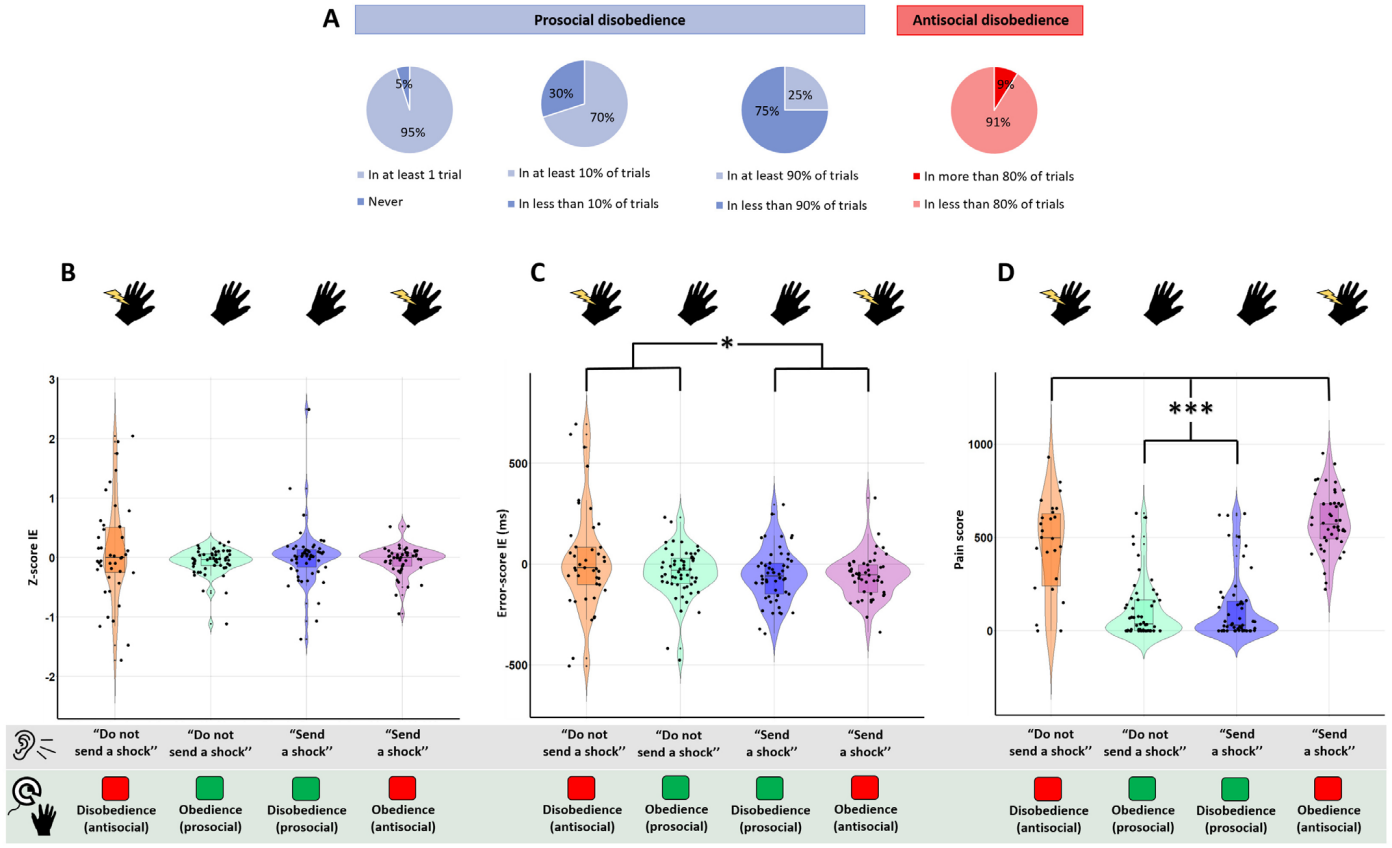
Behavioral results for the Agency and Empathy runs. Almost all agents disobeyed but at different degree, some of them being highly prosocial by not sending shocks despite being ordered to, whereas others adopting antisocial behaviors (A). In graphs B, C, and D, “Do not send a shock” and “Send a shock” indicate the auditory instructions received by the experimenter. Black dots represent individual values for each agent. Box-plots indicate the median and the interquartile range ± the standard deviation. Note that higher interval estimates in B and C (i.e., positive z-score or positive error-score) are associated with a lower SoA (less feeling to be the author of one’s own action), and reciprocally. Here, Instruction and Choice did not mainly influence interval estimates when measured by z-scores (B) or error-scores (C). In fact, a main effect of Instruction was only found for error-scores but mostly driven by antisocial disobedience trials. The “Do not send a shock”/Disobedience condition (orange) reflecting antisocial behavior showed the most variable responses. As expected, agents reported higher pain scores after sending a shock (orange, pink) than after not sending a shock to the victim (blue, green) (D) (*p< 0.05, ***p< 0.001).

Among the 56 included agents, only 3 for Agency and 1 for Empathy runs never refused to send a shock, thus leading to no disobedience. Seventy-five percent of agents in the Agency run and 71% of them in the Empathy run disobeyed in at least 10% of the trials where experimenter ordered to send a shock (i.e., prosocial disobedience, %Pro_disob). Fourteen agents in both runs reached more than 90% of prosocial disobedience trials (≥72/80 trials) (Fig. 2A). In order to better visualize prosocial disobedience over time, we also plotted individual curves showing cumulative prosocial disobedience across the 120 trials for each run. Overall, these plots (described inAppendix G) revealed three different profiles: (1) participants showing no prosocial disobedience (i.e., flat curve), (2) participants exhibiting a strong altruistic profile, almost consistently disobeying throughout the task (i.e., linearly increasing curve), and (3) participants showing more trial-by-trial choices, disobeying on certain trials (i.e., jagged curve). Moreover, agents were generally consistent across the two runs in their decision-making strategy.

The debriefing questions confirmed that agents disobeyed intentionally. Agents who never disobeyed explained that they just followed orders to correctly conduct the experiment. We also observed that five agents adopted an antisocial behavior by sending a shock, even when the experimenter did not order it, in more than 80% of the trials (i.e., antisocial disobedience) (Fig. 2A). They were excluded from*f*MRI analyses comparing obedience and disobedience trials due to their insufficient number of prosocial disobedience trials. We conducted a univariate ANOVA on the percentage of behavioral choice with Instruction (“Send a shock”; “Do not send a shock”), Choice (Obedience; Disobedience), and Run (Empathy; Agency) as factors. As expected, we found a main effect of Instruction with more shocks sent to the victim compared with no shock sent, but this may be due to the fact that the order to shock represented 67% of trials (mean “Send a shock” = 35.9%,$CI_{95}$: ±3.17%, mean “Do not send a shock” = 19.8%,$CI_{95}$: ±1.89%;*F*(1,332) = 75.05,*p*< 0.001,$\eta_{p}$² = 0.18,$BF_{incl}$> 100). The main effect of Choice was also significant with a greater obedience rate than disobedience rate (mean Obedience = 34.5%,$CI_{95}$: ±2.35%, mean Disobedience = 21.5%,$CI_{95}$: ±3.17%;*F*(1,332) = 62.41,*p*< 0.001,$\eta_{p}$² = 0.16,$BF_{incl}$> 100), without interaction with the Instruction factor (*F*(1,332) = 2.76,*p*= 0.1,$BF_{incl}$= 1.84). However, we did not find a significant main effect of Run nor interactions with the other factors, suggesting that participants disobeyed at the same extent in the two runs (all*p*’s > 0.50, all$BF_{incl}s$< 0.08). Moreover, we conducted a supplementary one-way ANOVA with Order of the run (Agency run first, Empathy run first) as between-subject factor to control for this effect. No order effect was found on the %Pro_disob suggesting that completing the Agency run first (or the Empathy run first) did not influence the agents’ choices (*p*= 0.77,$BF_{10}$= 0.28).Appendix Hreports the obedience and disobedience rates for each agent.

#### 3.1.2 Temporal binding

We conducted a repeated-measure ANOVA with two within-subject factors Instruction and Choice on IEs z-scores and error-scores (seeSection 2). We did not find significant main effects nor interactions on the z-scores with the frequentist approach (all*p*’s > 0.10) (Fig. 2B). Bayes factors corroborated these conclusions by showing evidence toward H0 (all$BF_{incl}$s < 0.30). However, error-scores revealed a main effect of Instruction (*F*(1,139) = 4.75,*p*= 0.03,$\eta_{p}$² = 0.03,$BF_{incl}$= 0.44) without other significant results (all*p*’s > 0.09, all$BF_{incl}s$< 0.70). Agents had higher positive error judgments, reflecting a lower SoA, when they received the order to not send a shock than to send a shock. This effect did not interact with the factor Choice, suggesting a similar effect regardless of their action outcomes. By looking visually at the distribution of the data, this effect seemed, however, mostly driven by the antisocial disobedience trials. When agents antisocially disobeyed, they indeed estimated longer delays (i.e., less SoA) than the three other conditions by showing negative mean error-scores (“Do not send a shock”/Disobedience: mean ±$CI_{95}$= 24.39 ± 77.53 ms, “Send a shock”/Obedience: -60.71 ± 30.65 ms, “Send a shock”/Disobedience: -57.39 ± 35.98 ms, “Do not send a shock”/Obedience: -42.70 ± 33.44 ms) (Fig. 2C).

#### 3.1.3 Subjective pain

We conducted the same repeated-measures ANOVA with Instruction and Choice as within-subject factors on the pain scale. We found main effects of Instruction (*F*(1,94) = 36.11,*p*< 0.001,$\eta_{p}$² = 0.28) and Choice (*F*(1,94) = 47.96,*p*< 0.001,$\eta_{p}$² = 0.34), as well as a significant interaction Instruction x Choice (*F*(2,94) = 228.61,*p*< 0.001,$\eta_{p}$² = 0.71). Those results were supported by the Bayesian approach showing strong pieces of evidence toward H1 for the same main and interaction effects (all$BF_{incl}$s > 100). As expected, agents reported higher pain scores after sending a shock to the victim (“Do not send a shock”/Disobedience: mean ±$CI_{95}$= 449.26 ± 82.46, “Send a shock”/Obedience: 586.86 ± 43.44) than when no shock was delivered (“Send a shock”/Disobedience: 123.1 ± 48.23, “Do not send a shock”/Obedience: 114.07 ± 43.5) (Fig. 2D), showing that they paid attention to what was displayed on the screen. Pairwise comparisons indicated that the two conditions leading to a shock did not differ between each other (*p*> 0.70,$BF_{10}$= 0.27). In the same way, the two conditions leading to a no shock did not differ between each other in terms of pain score (*p*> 0.70,$BF_{10}$= 0.18).

#### 3.1.4 Decision times (DTs)

For each run, we conducted the same repeated-measures ANOVA with Instruction and Choice as within-subject factors on DTs. No significant main effect nor interaction was found on DTs in both runs for the Instruction x Choice ANOVA (all*F*(1,139)s < 3.45, all*p*’s > 0.05,$BF_{incl}$s < 1).

#### 3.1.5 Correlations between behavioral measures

Results on IE did not reveal significant correlations between %Pro_disob and z-scores (*r*= 0.08,$p_{FDR}$= 0.59,$BF_{10}$= 0.18) nor error-scores (*r*= -0.24,$p_{FDR}$= 0.31,$BF_{10}$= 0.47). However, by controlling for the other types of obedience, a significant positive correlation appeared between the percent of obedience to send a shock and the z-score (*r*= 0.45,$p_{FDR}$= 0.01,$BF_{10}$= 765.56), suggesting that the more agents obeyed orders to send a shock, the lower their SoA was. The other correlations with the rate of antisocial disobedience or obedience to no shock were not significant ($p_{FDR}$s > 0.70,$BF_{10}s$< 3.70).

For empathy score, no significant correlation was found between %Pro_disob and pain scores (mean:*r*= -0.11,$p_{FDR}$= 0.42,$BF_{10}$= 0.52; median:*r*= -0.29,$p_{FDR}$= 0.097,$BF_{10}$= 0.53). We also conducted correlations between the %Pro_disob and the DT for both tasks, but all results were in favor of H0 ($p_{FDR}s$> 0.35,$BF_{10}$s < 0.35).

### 3.2 fMRI results at whole-brain level

As explained inSection 2, we computed four different contrasts (and the associated reverse contrasts) to target the epochs of our neurocognitive processes of interest during different phases of decision making (i.e., predecision, decision, and postdecision). In one contrast (i.e., [“Send a shock”/Obedience > “Send a shock”/Disobedience]), we focused specifically on trials involving immoral orders, that is, when the experimenter gave the orders to send a shock, and we contrasted trials where participants obeyed that order (i.e., antisocial obedience trials) or disobeyed that order (i.e., prosocial disobedience trials). In a second contrast, (i.e., [“Send a shock”/Obedience > “Do not send a shock”/Obedience]), we focused on obedience trials only and contrasted the order received. In a third contrast (i.e., [“Send a shock”/Obedience + “Do not send a shock”/Disobedience] > [“Do not send a shock”/Obedience + “Send a shock”/Disobedience]), we focused on the brain processing when victims received pain or no pain. Finally, we used a global contrast [“Send a shock” > “Do not send a shock”] to investigate the processing of the auditory instructions.Table 1inSection 2describes the processes targeted for each epoch of interest.

#### 3.2.1 Predecision phase and auditory processing

**Fig. 3.**
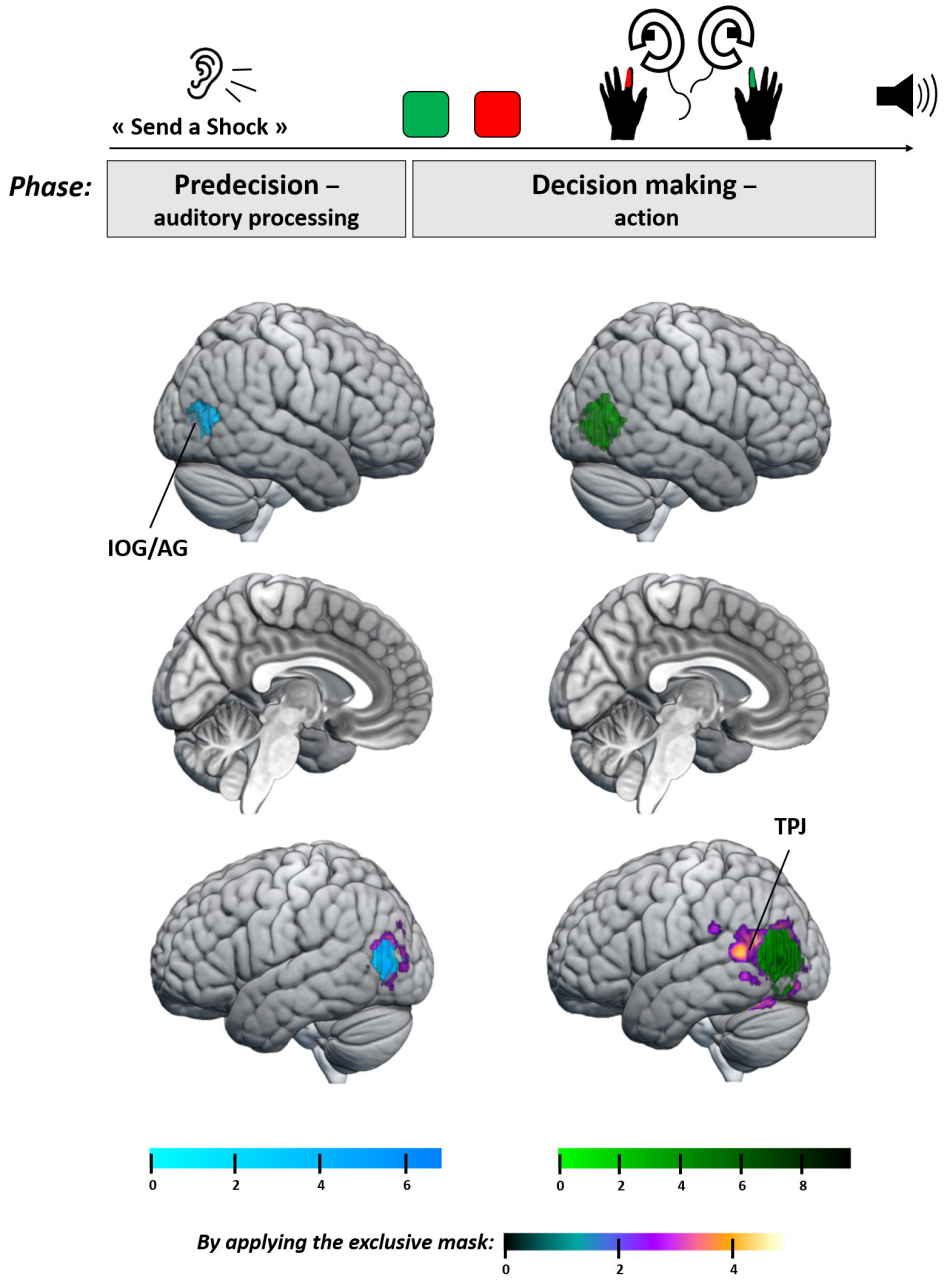
fMRI results at whole-brain level for the [“Send a shock”/Obedience > “Send a shock”/Disobedience] contrast (reflecting a similar immoral order received but a different behavior) at different time windows targeting the predecision phase for auditory processing (blue) and the decision-making phase for action (green). By applying an exclusive mask with clusters obtained for the [“Send a shock”/Obedience > “Do not send a shock”/Obedience] contrast, we focused on the disobedience effect, controlling for the shock delivery effect (purple to orange). Between auditory processing and the decision to obey and send a shock, agents activated more the left IOG/AG and left TPJ regions compared with when they decided to disobey.

**Fig. 4.**
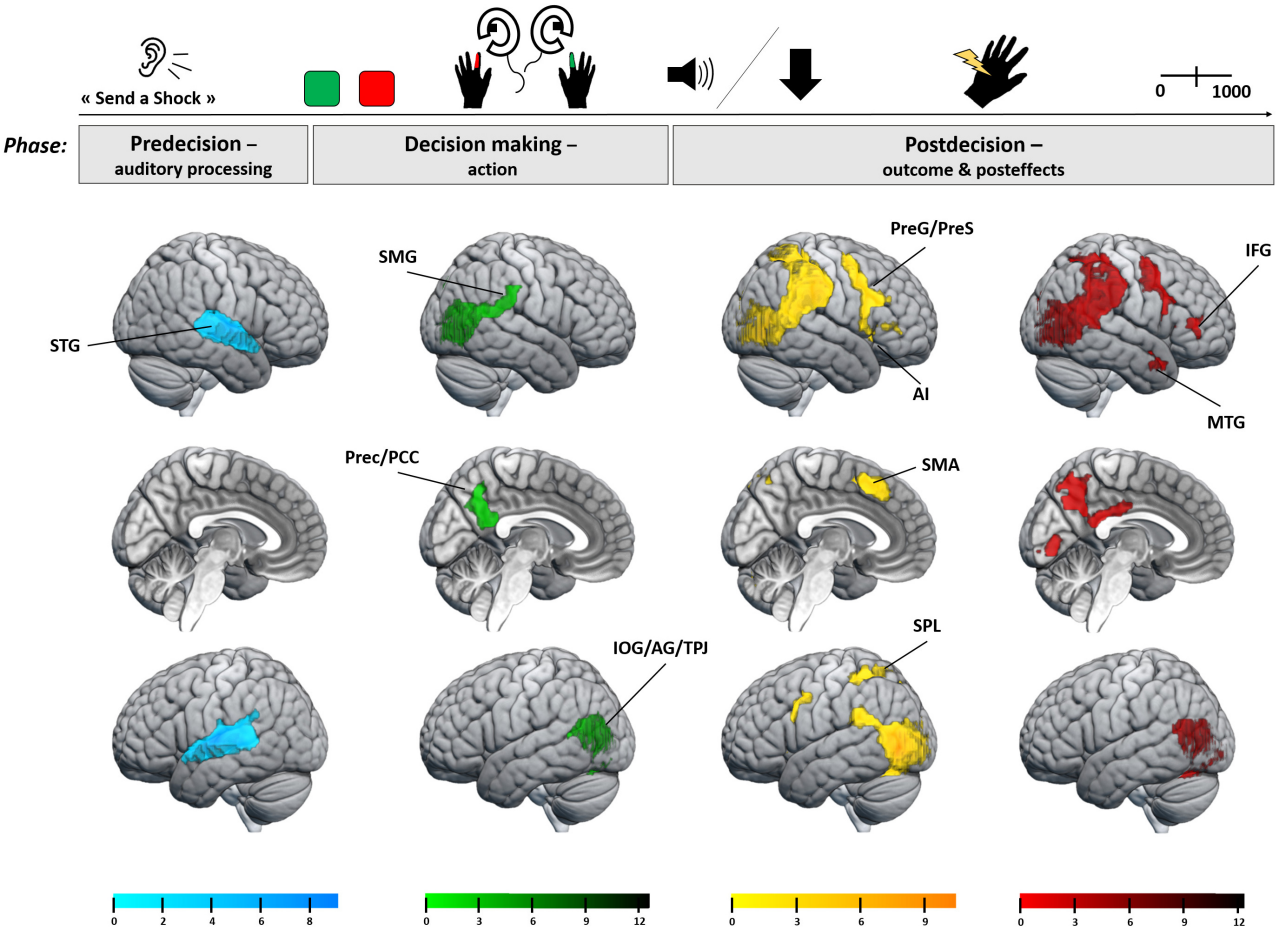
fMRI results at whole-brain level for the [“Do not send a shock” > “Send a shock”] instruction contrast at the time window targeting the predecision phase for auditory processing (blue), and for the [“Send a shock”/Obedience + “Do not send a shock”/Disobedience] > [“Do not send a shock”/Obedience + “Send a shock”/Disobedience] contrast (reflecting either the shock or the no shock received by the victim) at the three other time windows targeting the decision-making phase for action (green) and the postdecision phase for outcome (yellow) and posteffects (red). As a sanity check, agents activated more the auditory area (STG) when they received the longer “Do not send a shock” instruction compared with the “Send a shock” one (blue). When agents decided to send a shock, they activated more IOG/AG, TPJ, SMG, and Prec/PCC, compared with when they decided to not send a shock. After the victim received the shock, the network expanded to other frontoparietal areas, as well as the AI.

For this phase we used the 3,000 ms time window delay after the auditory instruction and before the keypress, during the Agency run. Using the [“Send a shock” > “Do not send a shock”] instruction contrast, we found no significant cluster. The reverse contrast revealed bilateral activation of the auditory areas ([-65 -13 6], Z = 7.13, cluster size = 1,429; [66 -7 6], Z = 6.27, cluster size = 778), an effect probably driven by the fact that the “Do not send a shock” instruction was longer to process than “Send a shock” instruction (Fig. 4). We used the [“Send a shock”/Disobedience > “Send a shock”/Obedience] contrast to investigate the changes between obedience and disobedience after an immoral order received. We only found significant clusters for the reverse [“Send a shock”/Obedience > “Send a shock”/Disobedience] contrast, with a modulation of bilateral inferior occipital gyrus extending to angular gyrus (Table 3;Fig. 3). By investigating the same contrast but applying the exclusive mask with significant clusters obtained for the [“Send a shock”/Obedience > “Do not send a shock”/Obedience] contrast, we found a similar cluster in the left hemisphere only ([-45 -79 18], Z = 5.19, cluster size = 328,*p*= 0.005). Bilateral IOG/AG was found using the [“Send a shock”/Obedience > “Do not send a shock”/Obedience] contrast (left: [-45 -79 9], Z = 5.56, cluster size = 313; right: [48 -67 6], Z = 5.74, cluster size = 387). Its reverse contrast revealed a significant cluster in bilateral auditory areas (left: [-58 -18 9], Z = 5.99, cluster size = 903; right: [57 -11 6], Z = 5.22, cluster size = 431).

**Table 3.** Significant clusters obtained using the [“Send a shock”/Obedience > “Send a shock”/Disobedience] contrast for the predecision and decision-making phases, targeting auditory and action processing reciprocally.

Anatomical location	MNI coordinates (in mm)			Z score	Cluster size (number of voxels)
[“Send a shock”/Obedience>“Send a shock”/Disobedience]	x	y	z		
Predecision phase for auditory processing					
Left Inferior Occipital Gyrus/Angular Gyrus	-49	-74	18	5.36	302
Right Inferior Occipital Gyrus/Angular Gyrus	52	-74	15	5.11	290
Decision-making phase for action					
Left Inferior Occipital Gyrus/Angular Gyrus	-52	-76	3	6.58	951
Right Inferior Occipital Gyrus/Angular Gyrus	52	-74	-4	6.20	507

The postdecision phase was not reported here because the contrast involved different visible outcomes based on different decisions (i.e., obey/disobey) that could not be dissociated, and was thus targeted in other contrasts.

#### 3.2.2 Decision-making phase and action processing

The decision-making phase was investigated during the Agency run with an epoch taken between the screen display of the Shock/No shock mapping where participants could see which button was associated with which action and the tone associated with the action’s outcome. Using the main [“Send a shock”/Obedience > “Send a shock”/Disobedience] contrast, we found a significant activation of bilateral IOG/AG extending to the TPJ (Table 3;Fig. 3). Even if larger, this cluster overlapped with the one found for the predecision phase. By adding the exclusive mask, this cluster remained in the left hemisphere only ([-61 -61 21], Z = 5.39, cluster size = 839,*p*= 0.005). Similar results were found bilaterally using the [“Send a shock”/Obedience > “Do not send a shock”/Obedience] contrast (left: [-52 -76 3], Z = 7.34, cluster size = 774; right: [48 -67 6], Z = Inf., cluster size = 1,459). No significant cluster was found for the reverse contrasts. We also identified clusters of the [“Send a shock”/Obedience + “Do not send a shock”/Disobedience] > [“Do not send a shock”/Obedience + “Send a shock”/Disobedience] contrast to investigate brain differences when agents decided to send a shock to the victim compared with when they did not. We found three clusters: one including the right IOG/AG, the TPJ, the dorsal part of the superior temporal gyrus (STG), and the SMG; a second one on the left hemisphere also including the IOG/AG and the TPJ; and a median cluster including the Prec/PCC (Table 4;Fig. 4). No significant cluster was found for the reverse contrast.

**Table 4.** Significant clusters obtained using the [“Send a shock”/Obedience + “Do not send a shock”/Disobedience] > [“Do not send a shock”/Obedience + “Send a shock”/Disobedience] contrast for three epochs: decision-making phase for action, and postdecision phase for outcome or posteffects.

Anatomical location	MNI coordinates (in mm)			Z score	Cluster size (number of voxels)
[“Send a shock”/Obedience + “Do not send a shock”/Disobedience] > [“Do not send a shock”/Obedience + “Send a shock”/Disobedience]	x	y	z		
Decision-making phase for action					
Left Inferior Occipital Gyrus/Angular Gyrus/Temporo-Parietal Junction	-49	-76	6	Inf	1,143
Right Inferior Occipital Gyrus / Angular Gyrus/Temporo-Parietal Junction/Superior Temporal Gyrus/Supramarginal Gyrus	48	-67	6	Inf	1,952
Precuneus/Posterior Cingulate Cortex	0	-47	21	4.53	316
Post-decision phase for outcome					
Left Inferior Occipital Gyrus/Angular Gyrus/Temporo-Parietal Junction/Superior Temporal Gyrus/Supramarginal Gyrus/Superior Parietal Lobule/Precuneus	-52	-74	-1	7.73	4,286
Right Inferior Occipital Gyrus/Angular Gyrus/Temporo-Parietal Junction/Superior Temporal Gyrus/Supramarginal Gyrus/Superior Parietal Lobule/Precuneus	41	-81	-13	7.24	5,845
Left Precentral Gyrus/Precentral Sulcus	-54	0	39	5.17	172
Right Precentral Gyrus/Precentral Sulcus/Inferior Frontal Sulcus/Inferior Frontal Gyrus	52	9	18	6.38	1,112
Left Anterior Insula	-34	18	9	4.82	140
Right Anterior Insula	41	21	-1	4.46	192
Supplementary Motor Area	-4	16	51	4.79	403
Pallidum/Putamen	-18	0	-7	4.03	135
Post-decision phase for posteffects					
Left Inferior Occipital Gyrus/Angular Gyrus/Temporo-Parietal Junction	-45	-76	6	7.80	2,287
Right Inferior Occipital Gyrus/Angular Gyrus/Temporo-Parietal Junction/Superior Temporal Gyrus / Supramarginal Gyrus/Superior Parietal Lobule / Precuneus/Posterior Cingulate Cortex	52	-70	-1	7.57	6,055
Right anterior Middle Temporal Gyrus	57	5	-22	5.15	137
Right Precentral Gyrus/Precentral Sulcus/Inferior Frontal Sulcus/Inferior Frontal Gyrus	48	0	57	5.01	901

The predecision phase was not targeted here because this epoch does not concern the period occurring after the decision targeted by this contrast.

#### 3.2.3 Postdecision phase and outcome processing

We targeted the postdecision phase for outcome processing during the Empathy run with a 1,500 ms time window corresponding to the display of the arrow pointing to the video of the victim’s hand receiving or not the shock. We analyzed the [“Send a shock”/Obedience + “Do not send a shock”/Disobedience] > [“Do not send a shock”/Obedience + “Send a shock”/Disobedience] contrast to isolate witnessing or not the victim’s pain, and we obtained huge clusters including bilateral IOG/AG, TPJ, STG, SMG, SPL, and Prec, but also bilateral PreG and precentral sulcus (PreS) extending to the right toward IFG, the bilateral AI, SMA, and basal ganglia with the left pallidum and putamen particularly (Table 4;Fig. 4). No significant cluster was found for the reverse contrast. Similar regions were observed using the [“Send a shock”/Obedience > “Do not send a shock”/Obedience] contrast and are detailed inAppendix C. This result was expected as this contrast also compared shock versus no shock outcomes.

Results obtained using the main [“Send a shock”/Obedience > “Send a shock”/Disobedience] contrast are given inAppendix I, but could not be interpreted, as it involved different visible outcomes (i.e., a shock vs. no shock) based on different decisions (i.e., obey/disobey) that could not be dissociated.

#### 3.2.4 Postdecision phase and posteffects

In both Agency and Empathy runs, during the postdecision phase we also investigated posteffects using the time window between the tone or the arrow display and the scale display (i.e., the interval estimate scale or the pain rating scale). Seeing the victim receiving a shock compared with not receiving it activated four regions in agents’ brain, similarly to the other epochs: bilateral IOG/AG extending to TPJ, as well as to STG, SMG, SPL, and Prec/PCC on the right hemisphere; right anterior middle temporal gyrus (MTG) and right PreG extending to PreS and inferior frontal sulcus (IFS) (Table 4;Fig. 4). No significant cluster was found on this contrast when interacting with the Run factor, suggesting a similar modulation of the networks in Agency and Empathy runs. Again and as expected, similar results were found using the [“Send a shock”/Obedience > “Do not send a shock”/Obedience] contrast and are detailed inAppendix C.

Results obtained for the main [“Send a shock”/Obedience > “Send a shock”/Disobedience] contrast are given inAppendix I.

### 3.3 Correlations between ROIs and prosocial disobedience (%Pro_disob)

ROIs were built based on the significant clusters obtained at whole-brain level focusing on the two contrasts investigating agent’s disobedience and obedience choices. We also added the vmPFC/ACC and dmPFC in this analysis as these two regions are part of ToM (Amodio & Frith, 2006;Preckel et al., 2018). For each epoch (predecision, decision making, postdecision for outcome and posteffects), we extracted the mean beta value of each of the 17 defined ROIs (seeTable 2) using the associated [“Send a shock”/Obedience > “Send a shock”/Disobedience] contrast and the [“Send a shock”/Obedience > “Do not send a shock”/Obedience] contrasts. The contrast [“Send a shock”/Obedience > “Send a shock”/Disobedience] was used to investigate whether a change of activity during the pre- and decision-making phases could result in different choices, leading to more or less prosocial disobedience rates. The contrast [“Send a shock”/Obedience > “Do not send a shock”/Obedience] was used to investigate whether agents maintaining brain activity during the postdecision phase after sending a shock, could be more susceptible to disobey. We also analyzed the two other phases for this contrast, with the hypothesis that processing more the auditory information or reducing their engagement during the decision-making phases of obedience to shock could lead to less disobedience. We then conducted multiple correlation analyses (Spearman tests with FDR correction) between the beta values and the %Pro_disob.

**Fig. 5.**
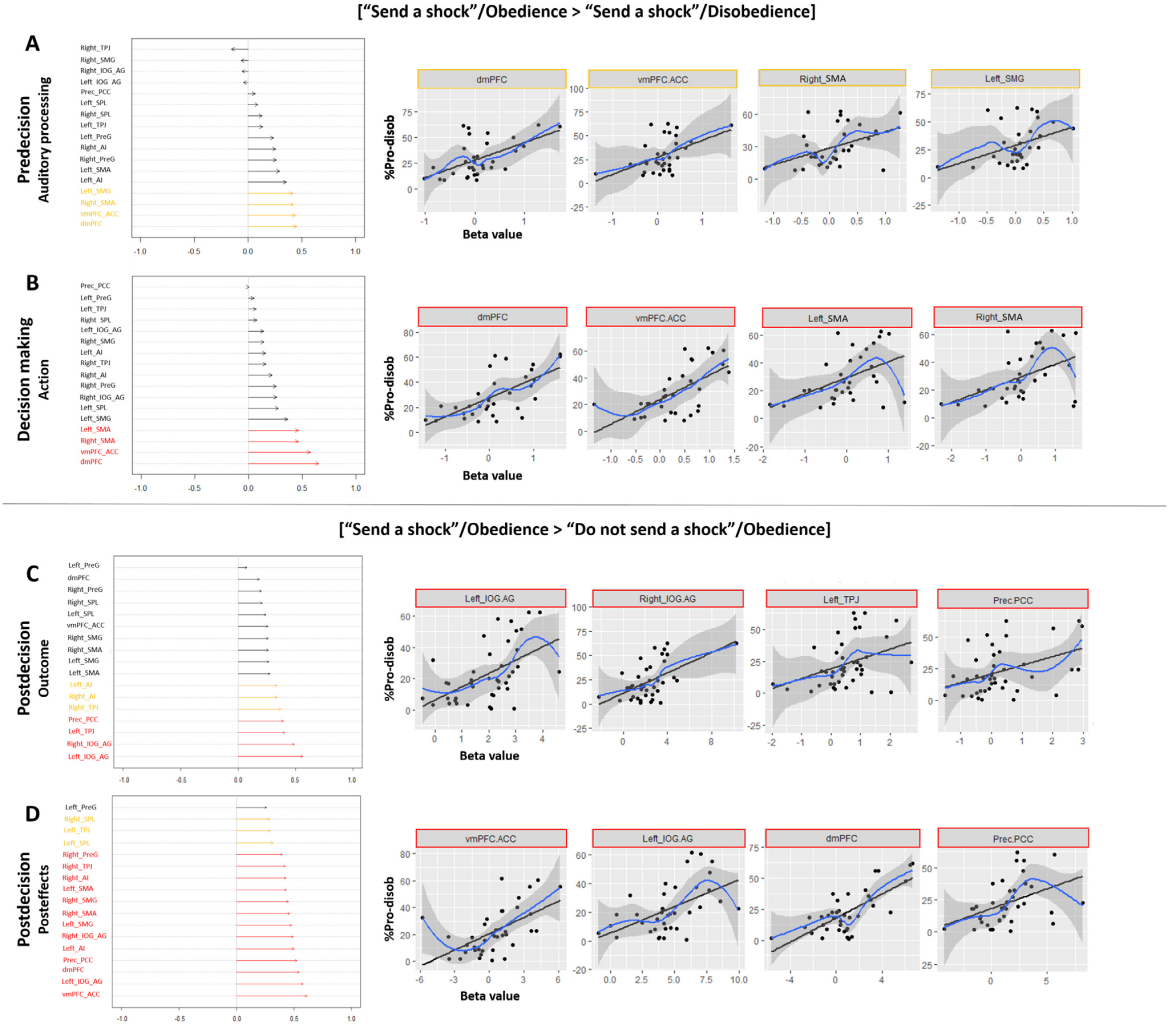
Graphical representations of the correlations. We observed multiple significative interactions between the mean beta values of the 17 ROIs and the %Pro_disob using the [“Send a shock”/Obedience > “Send a shock”/Disobedience] contrast for the predecision (A) and decision-making (B) phases, and the [“Send a shock”/Obedience > “Do not send a shock”/Obedience] contrast for the postdecision phase targeting the outcome (C) and the posteffects (D). Graphs on the left side report the correlation strength (*r*value) for each ROI, where arrows pointing toward the left indicate negative correlations and arrows pointing toward the right indicate positive correlations. Graphs on the right side represent the significant (red) or marginal (yellow) correlation curves between %Pro_disob and ROIs. Only the four mostly significant correlations for each epoch are reported in this figure. The other plots are given inAppendix J.

For the [“Send a shock”/Obedience > “Send a shock”/Disobedience] contrast targeting different behaviors for agents despite a similar order, we observed positive correlations between the %Pro_disob and the activity in several ROIs. During the predecision phase, these correlations were marginal for four ROIs: dmPFC (*r*= 0.45,$p_{FDR}$= 0.06), vmPFC/ACC (*r*= 0.44,$p_{FDR}$= 0.06), right SMA (*r*= 0.42,$p_{FDR}$= 0.06), and left SMG (*r*= 0.42,$p_{FDR}$= 0.06) (Fig. 5A;Appendix J). In the same way, the Bayesian approach indicated strong evidence toward H1 for dmPFC ($BF_{10}$= 48.89) and vmPFC/ACC ($BF_{10}$= 10.89) and moderate evidence toward H1 for bilateral SMA (left:$BF_{10}$= 2.41; right:$BF_{10}$= 3.29), and left SMG ($BF_{10}$= 2.18). During the decision-making phase, four ROIs were positively significantly correlated with %Pro_disob, a result supported by the Bayesian approach: dmPFC (*r*= 0.65,$p_{FDR}$< 0.001,$BF_{10}$= 712.31), vmPFC/ACC (*r*= 0.58,$p_{FDR}$= 0.004,$BF_{10}$= 252.91), and bilateral SMA (left:*r*= 0.46,$p_{FDR}$= 0.03,$BF_{10}$= 7.57; right:*r*= 0.46,$p_{FDR}$= 0.03,$BF_{10}$= 9.04) (Fig. 5B;Appendix J). During the postdecision phase, several positive correlations also emerged, and are detailed inAppendix J. Overall, results showed a positive association between activity in those ROIs (vmPFC, IOG/AG, and right PreG particularly for both epochs) and the %Pro-disob when participants refused to inflict harm to the victim. However, since the rate of prosocial disobedience differed between agents, the number of trials included to calculate ROI activity varied significantly, leading to variable signal-to-noise ratios. An additional analysis was conducted to address this limitation and is described inAppendix K. Overall, the findings replicated the results obtained for the predecision and decision-making phases, though significance did not remain during the postdecision period.

Using the [“Send a shock”/Obedience > “Do not send a shock”/Obedience] contrast targeting obedience trials only, we found positive correlations between the %Pro_disob and ROIs activity. Results for pre- and decision-making phases are described inAppendix Jand indicated overall that the higher the activity in vmPFC/ACC, bilateral IOG/AG, SMG, and TPJ when agent obeyed an order to send a shock, the more the %Pro_disob, or conversely. For the postdecision outcome period, four ROIs were found to positively correlate with %Pro-disob: bilateral IOG/AG (left:*r*= 0.56,$p_{FDR}$= 0.002,$BF_{10}$= 101.87; right:*r*= 0.49,$p_{FDR}$= 0.01,$BF_{10}$= 157.40), left TPJ (*r*= 0.40,$p_{FDR}$= 0.04,$BF_{10}$= 3.07), and Prec/PCC (*r*= 0.39,$p_{FDR}$= 0.04,$BF_{10}$= 5.93). The right TPJ (*r*= 0.37,$p_{FDR}$= 0.06,$BF_{10}$= 3.08) and bilateral AI (left:*r*= 0.34,$p_{FDR}$= 0.08,$BF_{10}$= 2.91; right:*r*= 0.34,$p_{FDR}$= 0.08,$BF_{10}$= 2.70) revealed marginal correlations (Fig. 5C;Appendix J). Finally, for the posteffects period, almost all ROIs were found significantly (or marginally) positively correlated with the %Pro-disob (*r*’s = [0.29; 0.61],$p_{FDR}$s = [0.08; <0.001],$BF_{10}$s = [1.32; >1,000]), with the exception of the left PreG (Fig. 5D;Appendix J), showing a positive relationship between brain activity in the postdecision phase and the number of times participants refused an order to harm the victim. Detailed statistics for each ROI and their associated plots are given inAppendix J.

We also conducted correlation analyses between ROIs activity and the IE z-scores and error-scores for the decision-making phase where SoA could occur, and the pain scores for the outcome period of the postdecision phase, using the difference between “Send a shock”/Disobedience and “Send a shock”/Obedience trials (Caspar et al., 2021). No significant correlation was found between the activity and the IE scores nor pain scores for any ROI (all$p_{FDR}$s > 0.7, all$BF_{10}$s < 0.6).

### 3.4 Investigation of the neurocognitive processes using NeuroQuery

**Fig. 6.**
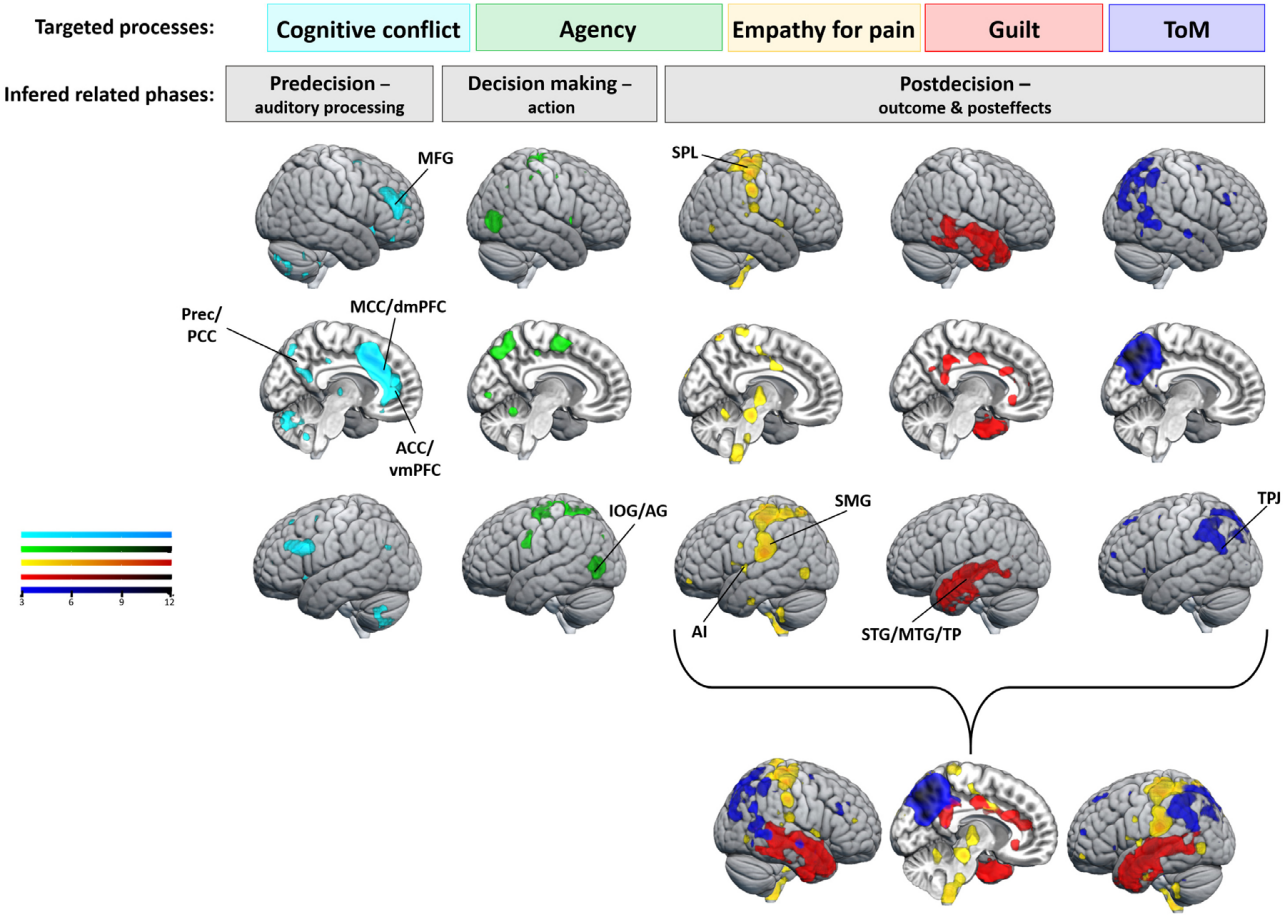
Maps generated by NeuroQuery using keywords related to our processes of interest: cognitive conflict (light blue), sense of agency (green), empathy for pain (combined with “hand” keyword on NeuroQuery) (yellow), guilt (red), and ToM (blue). We inferred these processes to occur during different decision phases. Particularly, by combining the three maps of empathy, guilt, and ToM, we obtained a similar map than the one found in our results for the postdecision phase.

To obtain a more reliable association between the observed activity in each decision phase with the targeted neurocognitive processes, we used NeuroQuery (Dockès et al., 2020). Based on our hypotheses, we focused on cognitive conflict, sense of agency, empathy for pain, feeling of guilt, and ToM processes. We entered these keywords in NeuroQuery to generate brain maps and to compare them with the maps obtained for each phase (predecision, decision making and postdecision) using the [“Send a shock”/Obedience > “Send a shock”/Disobedience] (Fig. 3) or the [“Send a shock”/Obedience + “Do not send a shock”/Disobedience] > [“Do not send a shock”/Obedience + “Send a shock”/Disobedience] contrasts (Fig. 4). We inferred that cognitive conflict could be involved between the predecision and the decision-making phase, as cognitive conflict typically occurs with the stimulus onset (Ito et al., 2019;Li et al., 2017;Van Veen & Carter, 2005), that is the auditory order in our case, and lasts until a decision is made (Caspar, Gishoma, et al., 2022). Then, choosing between following the experimenter’s order or following one’s own judgment by disobeying could lead to a conflict during this predecision phase (Götz et al., 2023;Mellers et al., 1998;Shafir et al., 1993). The map obtained by NeuroQuery using “cognitive conflict” keyword revealed the Prec, mPFC, cingulate regions, and the bilateral MFG (Fig. 6), being partially congruent with the obtained correlation results (Fig. 5A). The decision-making phase could represent SoA because it corresponds to the moment agents could start initiating their action and its consequence, thus representing a typical decision-making phase where agency can occur (Gallagher, 2000). Using the “agency” keyword, we obtained a map mainly composed of the bilateral IOG/AG region but also the Prec and the MCC (Fig. 6), very close to our results during the decision-making phase. Empathy for pain and feeling of guilt were expected to occur during the postdecision phase (Decety, 2011;Lamm et al., 2017). Whereas empathy could be more linked to the outcome, we inferred guilt to be related to posteffects, even if both processes should be involved in overlapped timelines. In NeuroQuery we used the “empathy” keyword along with the “pain” and “hand” keywords. It led to the map shown inFigure 6with several regions such as the bilateral AI, SPL, SMG, but also the brainstem (which was not investigated in our study). When using “guilt” as keyword, we mainly found bilateral temporal and cingulate regions. Finally, ToM could also be involved for this task, as mental state information is deemed necessary during moral decision-making and prosocial behaviors (Maliske et al., 2023;Ye et al., 2015;Young & Tsoi, 2013). We expected this network to be more activated during the postdecision phase, even if mentalization of the experimenter could also occur before the agent’s choice. “Theory of mind” keyword revealed a map composing of the Prec, as well as the bilateral TPJ, SPL, and MFG. When combining the three processes targeted during the postdecision phase, we obtained a comparable map with the one shown inFigure 4, but with extended temporal activity (Fig. 6).

### 3.5 Qualitative analyses

We explored the correlations between the %Pro_disob (for Agency and Empathy runs separately) and the subjective feelings, as well as the reasons for disobeying; rated by the agents during the debriefing phase. We did not find significant correlations with the subscales assessing subjective experiences (i.e., to feel responsible, to feel bad, to feel sorry, how painful the shocks were) ($p_{FDR}$s > 0.50;$BF_{10}s$< 0.50). However, concerning the reasons for disobeying (i.e., morality, sensitivity, shock quantity, money, entertainment, rejection of authority, experimental, fear of judgment, education, and country’s history criterions), a positive correlation was found for the morality criterion (“How much did you disobey for moral reasons?”) and the %Pro_disob (Agency run:*r*= 0.47,$p_{FDR}$= 0.006,$BF_{10}$= 28.94; Empathy run:*r*= 0.48,$p_{FDR}$= 0.004,$BF_{10}$= 73.83). A tendency was also found for the education criterion (“How much did your (familial) education influence your decision?”) and the rejection of authority criterion (“How much don’t you like being told what to do?”) (*r*’s = 0.31,$p_{FDR}$s = 0.07,$BF_{10}$s = [1.31; 3.50] for all correlations). A negative correlation was found with the money criterion (“How much did you want to win more money?”) (Agency:*r*= -0.44,$p_{FDR}$= 0.008,$BF_{10}$= 9.23; Empathy:*r*= -0.38,$p_{FDR}$= 0.03,$BF_{10}$= 4.15). Interestingly, there was also a positive correlation with the antisocial disobedience rate and the money criterion (Agency:*r*= 0.63,$p_{FDR}$< 0.001,$BF_{10}$> 10,000; Empathy:*r*= 0.53,$p_{FDR}$< 0.001,$BF_{10}$> 10,000) as well as the entertainment criterion (“How much did you want to make the experience more entertaining and less boring?”) (Agency:*r*= 0.51,$p_{FDR}$< 0.001,$BF_{10}$= 1,100.68; Empathy:*r*= 0.50,$p_{FDR}$= 0.001,$BF_{10}$= 1,529.39), and a negative correlation with the antisocial disobedience rate and the morality criterion (Agency:*r*= -0.49,$p_{FDR}$= 0.001,$BF_{10}$= 75.74; Empathy:*r*= -0.38,$p_{FDR}$= 0.02,$BF_{10}$= 39.75) as well as the sensitivity criterion (“How much did you feel bad for the victim?”) (Agency:*r*= -0.39,$p_{FDR}$= 0.01,$BF_{10}$= 2.21; Empathy:*r*= -0.35,$p_{FDR}$= 0.03,$BF_{10}$= 1.47).

Linear regressions were conducted as an exploratory analysis between the %Pro_disob and the questionnaires’ scores (ASC, AfM, MF, RPI, SD3, IRI) for each subscale. Our results indicated that prosocial disobedience was best explained by a model integrating the Power Prestige score of the AfM questionnaire. The higher the score in Power Prestige were, the less the prosocial disobedience (Agency:*t*= -3.08,*p*= 0.003, Empathy:*t*= -3.57,*p*< 0.001). RPI and IRI questionnaire subscores (i.e., Perspective taking and Fantasy) took also part of the model but did not reach significance (*p*> 0.06). The resulting models with all the associated statistics are detailed inAppendix B, together with the models found for antisocial disobedience rate.

Together these results indicated that agents refused to send a shock mainly for moral reasons, whereas several of them adopted an antisocial behavior for reducing their boredom but also for wining more money, particularly because they associate it with prestige and power in their daily life.

## 4 Discussion

The present study investigated the neural correlates of disobedience to immoral orders, focusing on three phases of the decision process: predecision with order processing, decision making with action, and postdecision with outcome and posteffects. Within these phases, we targeted two sociocognitive (cognitive conflict and SoA) and two socioaffective (empathy for pain and guilt) processes. Additionally, ToM was investigated as a third sociocognitive process, given its known involvement in moral decision-making and prosocial behaviors (Maliske et al., 2023;Ye et al., 2015;Young & Tsoi, 2013). Although the different phases allowed us to target specific processes, it is important to emphasize that the investigated mechanism of disobedience is part of a broader decision-making process, making it challenging to study each cognitive process in isolation, as they cannot be considered independently. Notably, several studies have for instance showed that losing the sense of responsibility, which is strongly linked to the sense of agency, for an observed pain reduced activity in the neural network associated with empathy for pain (Cui et al., 2015;Koban et al., 2013;Lepron et al., 2015) and reduces feelings of guilt (Yu et al., 2020).

Taking this into consideration, previous studies showed that several of these processes—notably the sense of agency, the feeling of responsibility, cognitive conflict, empathy for pain and guilt—were reduced when individuals follow orders compared with acting freely (Caspar et al., 2016,^2021^;Caspar, Ioumpa, et al., 2020;Caspar, Lo Bue, et al., 2020;Caspar & Pech, 2023). Further, the few studies conducted on resistance to immoral orders (Caspar, Gishoma, et al., 2022;Cheng et al., 2022) suggested that a greater attenuation of these processes when people follow an order results in a lower resistance to such orders. Building on these findings, our initial prediction was that disobedience could only occur if individuals maintained sufficient neural activity linked to the victim’s pain, and feelings of responsibility and guilt for causing them pain. We also expected that only individuals enhancing brain activity during their decision making, potentially associated with cognitive conflict with the experimenter’s order or with maintaining their SoA, would disobey.

The behavioral results revealed that nearly all agents demonstrated disobedience by resisting immoral orders, indicating the overall efficacy of the paradigm in inducing resistance to orders. However, distinct profiles emerged among the participants, indicating interindividual variability, in line with previous psychological studies (Bègue et al., 2015;Lepage et al., 2019). Approximately 25% of agents exhibited highly altruistic behavior, refusing to send a shock in over 90% of the trials, while about 30% decided to disobey immoral orders in less than 10% of the trials. Interestingly, 9% of agents adopted antisocial behavior by sending a shock even when not ordered by the experimenter, in more than 80% of the trials. In line with the study investigating prosocial disobedience using EEG on a population composed of Rwandese born after the genocide against the Tutsis (Caspar, Gishoma, et al., 2022), our observations indicated that agents primarily refrained from sending a shock due to moral reasons, as assessed by subjective questionnaires. Conversely, antisocial behavior was negatively correlated with moral and sensitivity criteria. The development of moral identity is shaped by various factors during development and is influenced by our environment and social interactions, resulting in interindividual variability (Krettenauer et al., 2022). But interestingly, even in distinct environments and cultures such as Rwanda and Belgium, the primary criterion influencing disobedience remains morality. For both populations, the more agents perceived the action of sending a shock to the victim’s hand as immoral, the more likely they were to refuse to do so.

Still at the behavioral level, we observed a positive correlation between the number of trials in which agents obeyed to send a shock and the IE’s z-score, showing that the more they sent shocks following an order, the lower their SoA was. This finding aligns with previous studies showing that obedience reduces SoA (Barlas, 2019;Caspar et al., 2016,^2017^) and that SoA is associated with moral behaviors (Caspar, Ioumpa, et al., 2022). However, we did not succeed to link prosocial disobedience rate with the implicit SoA, the pain scales, or the performance, as no correlation was found between %Pro_disob and IE scores, pain scores, nor DTs. Moreover, we did not find a significant effect of prosocial disobedience on these measures. Only an effect on the IE error-score suggested that agents who antisocially disobeyed estimated longer delays compared with agents who obeyed or prosocially disobeyed, indicating a lower implicit SoA. This implies that those who engaged in antisocial disobedience might have avoided feeling SoA for sending a shock when the experimenter did not request it. Implicit SoA has been previously linked to feeling of responsibility, but also to regret (Nicolle et al., 2011). It appears that agents adopting an antisocial behavior may not have experienced regrets, basing their decisions on factors other than moral considerations. Upon investigating the reasons for disobeying, we found that agents who adopted antisocial behavior did so to alleviate boredom and to earn more money, particularly because they associate it with prestige and power in their daily lives. This contrasts with prosocial disobedience, which exhibited a negative correlation with the money criterion. This suggests that agents deciding to adopt antisocial behavior may have done so for egoistic reasons, favoring their own reward (i.e., wining money, reducing boredom). This conclusion partially replicates the results of a previous study, where antisocial behavior positively correlated with the money criterion only (Caspar, Gishoma, et al., 2022). Except for the modulation of the IE error-score predominantly observed in antisocial behaviors, we did not detect a significant main effect of the instruction (“Send a shock”, “Do not send a shock”) on the SoA scores. Previous studies reported a reduced SoA when comparing a “coerced” condition, where actors were ordered, with a “free choice” condition, where agents decided independently whether to administer a shock (Caspar et al., 2016;Caspar, Lo Bue, et al., 2020). Here, an interpretation of disobedience could be that it involves a greater perception of freedom, by choosing a behavior independent of the one ordered by the experimenter. Following this reasoning, a greater temporal binding could have been observed when people chose to disobey orders compared with obeying orders. However, in those previous studies, the “free-choice” and “coerced” conditions were blocked, placing participants in one mindset or the other for several consecutive trials. In our study, actors received consistent instructions throughout the task, creating a continuous coercion effect and reducing the possibility to see a change in state-of-mind from one trial to another. A paradigm where participants must obey during several trials, and then disobey on other trials, might help to answer this pending question. It is also possible that disobeying orders, which may enhance the perception of freedom of choice, is balanced by the cognitive effort required to resist an order. Indeed, temporal binding has been found to be reduced when the cognitive effort required during a task is high (Howard et al., 2016).

No brain regions exhibited heightened activation in prosocial disobedience compared with obedience to send a shock across the three decision phases. However, the reverse contrast revealed a network evolving alongside the trial, primarily involving three clusters: a first one incorporating the bilateral IOG and AG; a second one including TPJ, SMG, and SPL bilaterally but more activated in the right hemisphere; and a last one including somatosensory and motor areas such as the PreG, PreS, and the SMA, and extending to the IFS. All these regions were more activated in obedience trials than in disobedience trials to send a shock. By excluding regions similarly activated in the comparison between obedience to send a shock and not sending a shock, we identified clusters that are specifically linked to disobedience, rather than simply reflecting the prosocial nature of the decision (i.e., choosing not to send a shock). The results showed left-lateralized regions, including the IOG/AG, TPJ, and SMG. These findings could suggest that while prosocial behavior is processed bilaterally in the occipitotemporoparietal intersection, the act of disobeying an order is more strongly associated with activity in the left hemisphere. One could extrapolate that this left-hemisphere preference may highlight the unique cognitive and social processes involved in challenging authority or overriding direct commands. Hemispheric asymmetries in the processing of moral judgments or prosocial decisions have already been observed in previous studies (Cope et al., 2010;Hecht, 2014;Hiraishi et al., 2021). They showed that judgment of immoral stimuli was preferentially processed in the left hemisphere, particularly in the left mPFC, TPJ, and PCC.

According to the dual-process hypothesis of moral judgment and decision making, improved brain activity observed during obedience versus prosocial disobedience may reflect two distinct processes: a fast, intuitive mechanism that facilitates prosocial responses and a slower, more rational process that notably involves emotional regulation and leads to obedience even in morally ambiguous situations (Haidt, 2001;Haidt & Bjorklund, 2007). However, this interpretation should be approached with caution. Our behavioral data reveal no significant impact of decision type on either the timing or the trajectory of decisions across trials (seeAppendix L), challenging the applicability of this dual-process model to our experimental design. Moreover, previous research under this framework suggests that prosocial, intuitive responses are more likely when decision time is constrained (Rand et al., 2012,^2014^). In our study, participants were afforded “unlimited” time to make their decision, complicating any straightforward classification of their decisions as either intuitive or rational.

Surprisingly, contrary to hypotheses, particularly for SoA and cognitive conflict, no (pre)frontal or cingulate regions were found to be modulated at the whole-brain level. The identification of the IOG/AG across all epochs could suggest a key role in distinguishing between sending a shock or not. Previous research has shown involvement of the AG during moral judgment tasks (Raine & Yang, 2006). A disruption of this area was also found to be associated with antisocial behaviors (Raine & Yang, 2006). Finally, increased AG activity was previously found to be linked to reduced SoA (Beyer et al., 2018). The increased activity in the left side of this region observed for obedience compared with disobedience could indicate that agents enhanced their moral judgment about the received order to shock. By obeying this order, they also could reduce their SoA, in line with previous findings (Caspar et al., 2016,^2018^;Caspar, Lo Bue, et al., 2020). This effect persisted after the decision, suggesting a continuous involvement of these processes. As similar activity in the right AG was found when comparing shock versus no shock outcomes, it suggests that agents may differ in their moral judgment according to the visual outcome, even when this moral judgment has not been enough to prevent them from sending a shock during the decision phase. Moreover, AG extended to the TPJ from the decision-making phase. Identifying such activity during this decision-making time window is interesting as TPJ has also been associated with moral judgment and moral decision making, particularly in real scenarios (Feldman Hall et al., 2012). Ye and collaborators indeed used tDCS on bilateral TPJ and showed that a positive or negative modulation of this area’s activity led to a change in the moral judgment (Ye et al., 2015). However, the design of this previous study (i.e., a cathodal stimulation on the left TPJ simultaneously with an anodal stimulation on the right TPJ, or reversely) did not allow to clearly establish the direction of this effect. Together AG and TPJ were associated with the other network (as opposed to the self-network which includes ACC and AI), related to socially oriented processing and contextual information integration (Murray et al., 2015). In a study virtually replicating Milgram’s experiment, reduced activity in the right TPJ (with cathodal stimulation) was linked to a lower reluctance to cause harm to others (i.e., avatar) (Cheng et al., 2022). It suggested that decreased activity in TPJ, indicating an impaired capacity to take the victim’s perspective, could lead to higher obedience to send a shock, whereas maintained activity in this region could facilitate disobedience. Future analyses should explore how AG connects with other regions, including empathy- and guilt-related areas and the self-network, to better understand the role of AG in the disobedience process. In a meta-analysis, coactivation between AG and regions associated with the “social brain” (ToM and empathy networks) has been described at rest (Maliske et al., 2023). In our paradigm, a positive coupling between AG and the social brain during disobedience compared with obedience could express increasing consciousness toward the victim.

The postdecision phase, which includes outcome processing and posteffects, revealed increased joint activity in several brain regions (i.e., SMG, TPJ, somatosensory areas, IFG, intraparietal lobule, AI, Prec/PCC, basal ganglia, and MTG) when participants delivered a shock compared with when they did not. Based on our qualitative analysis using NeuroQuery, these regions could refer to empathy, guilt, and Theory of Mind networks—all of which are components of the social brain (Maliske et al., 2023;Reniers et al., 2012,^2014^). Joint activity of these networks has been observed when individuals face complex social situations (Preckel et al., 2018). In the present study, the social situation could be defined as complex because different actors were involved, and participants had to decide whether to follow the experimenter’s orders, regardless of the pain inflicted on the victim, or to act inversely. More specifically, increased activity in the SMG and TPJ has been shown to suggest an engagement in self-other distinction, facilitating perspective taking and reducing egocentricity (Lamm et al., 2017;Preckel et al., 2018). The SMG, in conjunction with somatosensory areas such as the PreG, PreS, and SMA, is known to play a central role in empathy for pain and feelings of guilt (Bastin et al., 2016,^2021^;Caspar, Ioumpa, et al., 2020;Preckel et al., 2018). Additionally,Caspar, Ioumpa, et al. (2020)found heightened activity in the insula, ACC, basal ganglia, MTG, and TPJ when participants observed shocks being administered compared with no-shock outcomes. Considering this literature, the increased activity in these regions during the postdecision phase likely reflects the engagement of ToM, empathy, and guilt networks in the present study. This heightened activity could be interpreted as the result of either enhanced processing of the experimenter’s perspective via ToM-related areas or increased processing of the victim’s pain through empathy and guilt networks. However, the similarity between the NeuroQuery map generated using the keywords “empathy,” “guilt,” and “ToM” and the neural maps we obtained from our contrasts in the postdecision phase suggests stronger support for the victim’s pain processing hypothesis. This interpretation aligns with our behavioral data, where participants reported higher pain scores after witnessing a shock delivered to the victim’s hand.

Too many agents were discarded when considering the global model with the contrast [(“Send a shock”/Disobedience> “Do not send a shock”/Disobedience) > (“Send a shock”/Obedience > “Do not send a shock”/Obedience)], leaving us unable to include it in the analyses. This analysis would have revealed solely brain regions related to the disobedience effect, by controlling for the witnessed shock effect. The spontaneous character of agent’s decision in our paradigm did not allow us to control the number of each type of obedience and disobedience, restricting the types of analyses we could conduct effectively. Behavioral variability is inherent to human studies, and obtaining a low antisocial disobedience rate indicates that our participants chose mostly not to engage into antisocial conducts against the experimenter’s instructions. This low antisocial disobedience rate is consistent with previous studies (Caspar et al., 2021;Caspar, Gishoma, et al., 2022). Obtaining a similar number of trials for each behavior would request removing freedom of choice and asking participants to disobey antisocially as part of the task’s instructions on certain fixed trials. However, this approach would hinder a reliable investigation of the mechanisms of disobedience, as obeying or disobeying would then be part of the instruction.

Correlation analyses between ROI activity and %Pro_disob indicated that, overall, the higher the prosocial disobedience, the greater the activity in ROIs when sending a shock compared with not sending a shock. Prior to the outcome associated with decision making, this effect was particularly evident in dmPFC, vmPFC/ACC, and bilateral SMA. Although this analysis is limited by varying signal-to-noise ratios due to interindividual differences in decisions, which could influence the results, these findings can still be cautiously interpreted in light of the existing literature. The positive modulation suggests that an engagement of frontal and prefrontal median regions before to send a shock helps agents to resist immoral orders. However, as this is a correlational approach, it is also possible that those who resist immoral orders enhance more frontal and prefrontal median regions. Notably, these regions play a crucial role in the decision-making process and cognitive control (Amodio & Frith, 2006;Li et al., 2017;Menon & D’Esposito, 2022), possibly revealing that an engagement of these areas may correspond to agent’s cognitive conflict when the experimenter asks them to send a shock. This is in line with the previous EEG study indicating that the higher the cognitive conflict before to send a shock, as revealed by higher midfrontal theta activity, the higher the general willingness to resist immoral orders (Caspar, Gishoma, et al., 2022). Moreover, dmPFC and vmPFC play a key role in social cognition by integrating the knowledge and point of view of others in contrast to the self’s perspective. They also encode values and rewards associated with others’ choices (Amodio & Frith, 2006;Bicks et al., 2015;Preckel et al., 2018). When these regions are activated, social components are processed as a priority, considering others’ choices and influencing decision making (Nook & Zaki, 2015). Conversely, reduced activity in vmPFC has been associated with selfish choices (Strombach et al., 2015). This might suggest that modulating activity in these regions could help in disengaging from the experimenter, allowing agents to focus on their personal moral judgments and ultimately disobey by refusing to send a shock. This hypothesis will require specific validation in future studies.

Finally, during the postdecision phase, we observed positive correlations between %Pro_disob and regions more activated during obedience to send a shock than no shock. These regions include and encompass empathy- and guilt-related brain regions, such as Prec/PCC, mPFC, TPJ, SPL, SMG, SMA, AI, or AG. It suggests that agents who strongly distinguished between sending a shock or not when obeying orders, as reflected by activity in this social brain network, were more likely to disobey and resist sending pain to the victim. This result is in line with a previous study showing that individuals who maintained their neural response to the pain of others when obeying orders were more likely to refuse sending a shock (Caspar, Gishoma, et al., 2022). However, as this is a correlational approach, it may also be the case that those who have a greater prosocial disobedience also engage more the brain regions involved in the processing of the victim’s pain. Importantly, we cannot rule out that the activity in the observed regions might reflect other cognitive processes such as attentional focus or the level of effort and engagement in the task. We could expect that the more agents paid attention to the task, the higher their brain activity would be during the postdecision period to process the outcome, potentially influencing their prosocial disobedience in subsequent trials. However, our behavioral measures (detailed inAppendix L) do not appear to support this attention-related hypothesis. Furthermore, the contrasts of interest revealed no differences in the visual areas, further suggesting that attention levels remained consistent across conditions. Concerning the hypothesis of varying effort levels among agents, the prediction would be that the easier it is for an agent to obey—meaning they feel no particular reason to disobey—the less cognitively demanding and effortful the task would be perceived, resulting in reduced brain activity compared with individuals with high rates of disobedience. The experimental paradigm developed in this study does not allow us to conclusively support or refute this hypothesis, paving the way for future research.

Future studies should then build upon these initial findings on prosocial disobedience and delve deeper into the mechanisms underlying the switch from obedience to disobedience to send a shock. It is plausible that a pronounced neural attenuation of key neurocognitive processes involved in decision making during obedience could lead to a heightened challenge to switch toward resistance to coercion and ultimately disobedience. When deciding whether to obey or not, individuals must consider various pieces of information in their environment, including the victim’s pain, their own judgment compared with what is asked of them, and the perceived responsibility and guilt associated with obeying versus disobeying. We hypothesized that only if individuals maintain sufficient neural activity of these socioaffective and sociocognitive processes, especially when causing pain to someone else, can they ultimately resist and defy the order. In an exploratory analysis, we compared the obedience to send a shock trial at time*t*that led to prosocial disobedience*versus*obedience to send a shock during the next trial at time*t*+ 1. We particularly expected finding higher brain activity at time*t*during the postdecision phase, when agents disobeyed in the following trial at time*t*+ 1, corroborating the correlation results. While our exploratory results did not reveal significant brain regions, the limited number of trials in each condition may have lacked sufficient power to draw conclusions. Increasing the sample size or the number of trials could enhance the probability of obtaining enough trials in each condition for similar analyses. However, we did not observe high intraindividual variability, suggesting that participants maintained consistent behavior throughout the session—either consistently prosocial, antisocial, or obedient. This consistency prevented us from conducting multiple within-subject analyses.

This study represents the first investigation of the associated brain networks involved in prosocial disobedience using fMRI. Summarized inFigure 7, based on our findings and those obtained in previous studies (Caspar et al., 2021;Caspar, Gishoma, et al., 2022;Caspar, Ioumpa, et al., 2020), we propose that when agents receive experimenter’s instructions to send a shock, only those who engage AG and median prefrontal areas to maintain their cognitive conflict between self and other, and those who enhanced their SoA, ultimately focus on their own moral judgment and disobey. When focusing on the postdecision phase, empathy- and guilt-related brain areas were recruited only if a shock occurred, that is, when they witnessed a shock. Therefore, maintaining this brain activity when witnessing a shock following an order could favor participants to disobey more. ToM was also involved, although the present study did not allow for discrimination between the experimenter’s or victim’s perspective. Future studies will need to test this proposed timeline more precisely. Moreover, since this study investigated a specific type of disobedience under precise experimental circumstances, it is possible that different mechanisms may be involved in other contexts of disobedience. For instance, other forms of disobedience might rely on different processes.

**Fig. 7.**
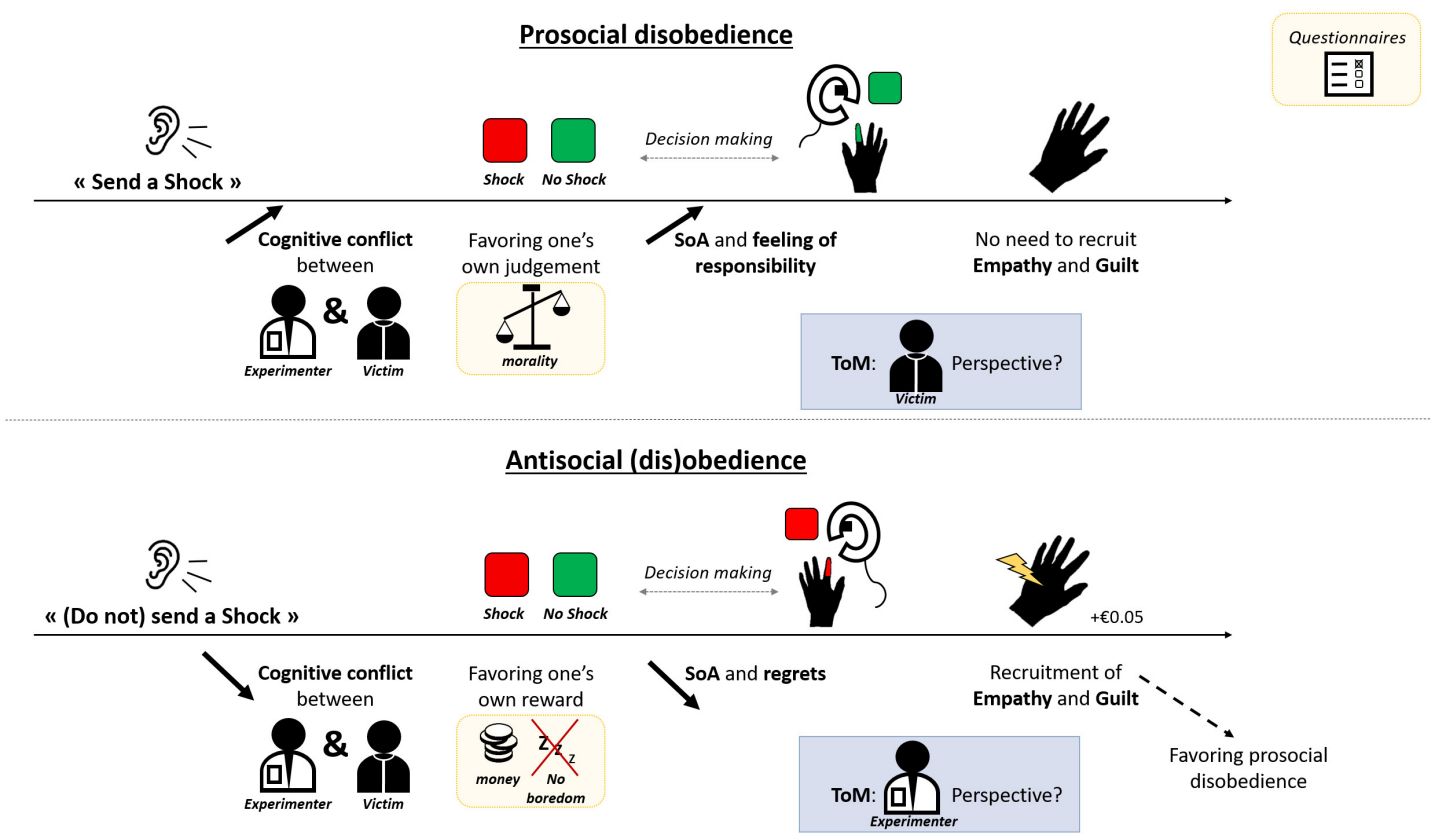
Schematic representation of the proposed timeline of involved processes which could lead to either prosocial disobedience or antisocial disobedience and obedience to send a shock.

As this study demonstrates, resisting the orders of an authoritative figure could involve complex cognitive and emotional processes. These processes highlight the intricate interplay between individuals’ decision making, the influence of authority figures, and the experience of the victim’s pain. By utilizing techniques such as hyperscanning and manipulating social contexts, future investigations can address whether prosocial disobedience primarily stems from disengagement from the experimenter, engagement toward the victim, or a combination of both factors. A critical avenue for future research involves understanding the relative importance of these processes and identifying interventions that target them effectively. Instances of damaging obedience persist worldwide, leading to significant suffering. It is imperative to identify strategies that empower individuals to resist such orders and mitigate the occurrence of such atrocities.

## Data and Code Availability

Data are made available on OSF (https://osf.io/xcgak/).

## Author Contributions

L.T. designed the study; conducted experiments; preprocessed, modeled, and analyzed data; and wrote the manuscript. A.R. performed experiments and helped preprocessed, modeled, and analyzed data. E.C. designed and led the study and wrote the manuscript. All the authors read and edited the manuscript.

## Declaration of Competing Interest

The authors declare no competing interest.

## Supplementary Material

Supplementary Material

## Supplementary Materials

Supplementary Appendixes for this article is available with the online version here:https://doi.org/10.1162/imag_a_00392
