# Supplementary material for: When the brain says “No!”: An MRI study on the neural correlates of resistance to immoral orders
Source: Imaging Neurosci (Camb). 2024 Dec 19;2:imag-2-00392. doi: 10.1162/imag_a_00392 (PMC12315749; doi:10.1162/imag_a_00392)
Supplement: Supplementary Material [file imag_a_00392-supp.pdf]

Appendices

When the brain says “No!”: An MRI study on the neural correlates of resistance to immoral orders.

Tricoche, L.[1], Rovai, A.[2], Caspar, E. [1]*

*corresponding author, emilie.caspar@ugent.be

[1]: affiliated to the Moral & Social Brain Lab, Department of Experimental Psychology, Ghent University, Belgium.

[2]: affiliated to the Translational Neuroanatomy and Neuroimaging Lab, Université Libre de Bruxelles, Belgium.

Appendix A

The debriefing phase, which was completed by the agents at the end of the experiment to investigate their subjective feeling of responsibility and their reasons for disobeying or obeying. Agents were also asked to estimate the number of trials they disobeyed (prosocially or antisocially) and how they felt during the experiment by open-ended question.

1. How responsible did you feel when you were the agent?

Not at all ―――――――――――――――――――――――――――― Extremely

2. How bad did you feel when you delivered a shock in exchange of money?

Not at all ―――――――――――――――――――――――――――― Extremely

3. How sorry did you feel when you delivered shocks to the 'victim'?

Not at all ―――――――――――――――――――――――――――― Extremely

4. Please rate how painful do you think the shocks were for the victim?

Not at all ―――――――――――――――――――――――――――― Extremely

5. Did you intentionally disobey?

YES **NO**

- If you indicated **NO** in the previous question, can you explain your reasons for following the orders throughout the experiment?

____
____
____
____
____
____
____

- If you indicated **YES** in the previous question, please answer the following questions about the reasons for disobeying.

A. I felt bad for the victim.

Not at all ―――――――――――――――――――――――――――― Extremely

B. There were too many shocks.

Not at all ―――――――――――――――――――――――――――― Extremely

C. I don't like being told what to do.

Not at all ―――――――――――――――――――――――――――― Extremely

D. I wanted to make more money.

Not at all ―――――――――――――――――――――――――――― Extremely

E. I was bored and it was a way to make it more entertaining.

Not at all ——— Extremely

F. I thought it was the aim of the experiment.

Not at all ——— Extremely

G. I was afraid to feel judged by the other persons present in the room.

Not at all ——— Extremely

H. My (family) education influenced my decisions.

Not at all ——— Extremely

I. The history of my country influenced my decisions.

Not at all ——— Extremely

J. For moral reasons.

Not at all ——— Extremely

- Can you estimate the number of trials you refused to deliver a shock?
- Can you estimate the number of trials you refused to NOT-deliver a shock?

6. If you disobeyed for other reasons please explain here:

____
____
____
____
____
____

7. Please describe in a few words how did you feel during the experiment:

____
____
____
____
____

Appendix B

Methodology and results of the linear regressions analyses conducted to investigate which individual characteristics, assessed by questionnaires, could the best explain the prosocial and antisocial disobediences.

Linear regressions were conducted as an exploratory analysis between the %Pro_disob and the questionnaires' scores (ASC, AfM, MF, RPI, SD3, IRI). All sub-scores of each questionnaire were integrated in the analysis. We conducted both forward and backward stepwise regressions to select the best model according to the AIC, for each Agency and Empathy runs. For Agency run, the best model included four factors (AIC=317.57): Power Prestige of AfM (coef=-1.49), Perspective taking of IRI (coef=-1.26), Fantasy of IRI (coef=-0.97) and RPI score (coef=13.32). The linear regression analysis on this model only revealed a significant effect of the Power Prestige of AfM (t=-3.08, p=0.03; Perspective taking of IRI: t=-1.89, p=0.06; Fantasy of IRI: t=-1.59, p=0.11; RPI: t=1.74, p=0.09). Similarly, for Empathy run, the best model included three factors (AIC=316.27): Power Prestige of AfM (coef=-1.44), Perspective taking of IRI (coef=-1.06) and Fantasy of IRI (coef=-0.95). The linear regression analysis on this model revealed a significant effect of the Power Prestige of AfM (t=-3.57, p=0.001; Perspective taking of IRI: t=-1.61, p=0.11; Fantasy of IRI: t=-1.58, p=0.12).

We also explored which individual characteristics could explain the antisocial behavior (using the antisocial disobedience rate). For Agency run, the best model included six factors (AIC=218.48): Narcissism of SD3 (coef=0.94), Machiavelism of SD3 (coef=-0.42), Psychopathy of SD3 (coef=-0.37), Power Prestige of AfM (coef=0.71), Personal distress of IRI (coef=0.43) and Harm of MF (coef=-0.78). The linear regression analysis showed a significant effect of the Power Prestige of AfM (t=3.82, p<0.001), the Narcissism of SD3 (t=3.83, p<0.001) and the Harm of MF (t=-2.16, p=0.04). The other factors were non-significant (all p's>0.10). Finally, for Empathy run, the best model included three factors (AIC=222.71): Power prestige of AfM (coef=0.25), Narcissism of SD3 (coef=0.54) and Fairness of MF (coef=-0.83). Only the Narcissism of SD3 and the Fairness of MF were significant (Narcissism of SD3: t=2.48, p=0.02; Fairness of MF: t=-2.35, p=0.02; Power Prestige of AfM: t=1.64, p=0.11).

According to these results, the Power Prestige sub-score of AfM is the main factor explaining the disobedience behavior, with a negative relationship with prosocial disobedience and a positive relationship with antisocial disobedience. It suggests that individuals associating the most money with prestige and power in their daily-like were those adopting more an antisocial behavior; whereas individuals who did not associate money this prestige and power mostly adopted a prosocial behavior.

Appendix C

Results on the three contrasts analyzed as control in the present study. A similar network was found between the main [“Send a shock”/Obedience > “Send a shock”/Disobedience] contrast and the control [“Send a shock”/Obedience > “Do not send a shock”/Obedience] contrast. Its suggests that the effect mainly refers to the witnessed shock outcome, particularly for the post-decision phase.

- The [“Do not send a shock”/Disobedience > “Send a shock”/Obedience] contrast as well as the reverse contrast did not reveal any significant cluster whatever the phase (pre-decision, decision-making, post-decision for outcome or post-effects). This absence of significant result could be due to the few numbers of participants included in this analysis (N=19).
- The [“Send a shock”/Disobedience > “Do not send a shock”/Obedience] contrast did not reveal any significant cluster whatever the epoch. However, the reverse contrast showed increased activity in bilateral auditory areas for the pre-decision phase ([-67 -9 6], Z = 6.63, cluster size = 1098; [59 -13 9], Z = 5.26, cluster size = 497).
- As illustrated by the figure bellow, the [“Send a shock”/Obedience > “Do not send a shock”/Obedience] contrast showed increased activity in bilateral AG, for all epochs. For the post-decision phase, increased activity was also found in bilateral TPJ, SPL, SMG, PreG, left AI, SMA and PCC/MCC for the outcome period; and in right TPJ, right SPL, right SMG, right PreG, as well as Prec/PCC for the post-effects period. All coordinates and statistics are given in the following table. The reverse contrast only revealed significant activity in bilateral auditory areas during the pre-decision phase ([-58 -18 9], Z = 5.99, cluster size = 903; [57 -11 6], Z = 5.22, cluster size = 431).

Anatomical location	MNI coordinates (in mm)			Z score	Cluster size (number of voxels)
[“Send a shock”/Obedience >”Do not send a shock”/Obedience]	x	y	z		
Pre-decision phase for auditory processing					
Left Inferior Occipital Gyrus / Angular Gyrus	-45	-79	9	5.56	313
Right Inferior Occipital Gyrus / Angular Gyrus	48	-67	6	5.74	387
Decision-making phase for action					
Left Inferior Occipital Gyrus / Angular Gyrus	-52	-76	3	7.34	774
Right Inferior Occipital Gyrus / Angular Gyrus	48	-67	6	Inf	1459

Post-decision phase for outcome					
Left Inferior Occipital Gyrus / Angular Gyrus / Supramarginal Gyrus / Temporo-Parietal Junction	-52	-74	-1	7.34	2714
Right Inferior Occipital Gyrus / Angular Gyrus / Supramarginal Gyrus / Temporo-Parietal Junction / Superior Parietal Lobule	48	-74	9	6.51	5603
Left Superior Parietal Lobule	-29	-56	57	4.94	633
Left Precentral Gyrus	-52	0	39	4.83	216
Right Precentral Gyrus / Inferior Frontal Sulcus	52	9	18	5.55	1147
Left Anterior Insula / Supplementary Motor Area / Middle and Posterior Cingulate Cortex	-31	25	-7	4.71	197
Post-decision phase for post-effects					
Left Inferior Occipital Gyrus / Angular Gyrus / Temporo-Parietal Junction	-45	-76	6	7.46	1799
Right Inferior Occipital Gyrus / Angular Gyrus / Temporo-Parietal Junction / Supramarginal Gyrus / Superior Parietal Lobule / Precuneus / Posterior Cingulate Cortex	52	-70	-1	7.15	4859
Right Precentral Gyrus / Inferior Frontal Sulcus	45	-2	51	4.81	666

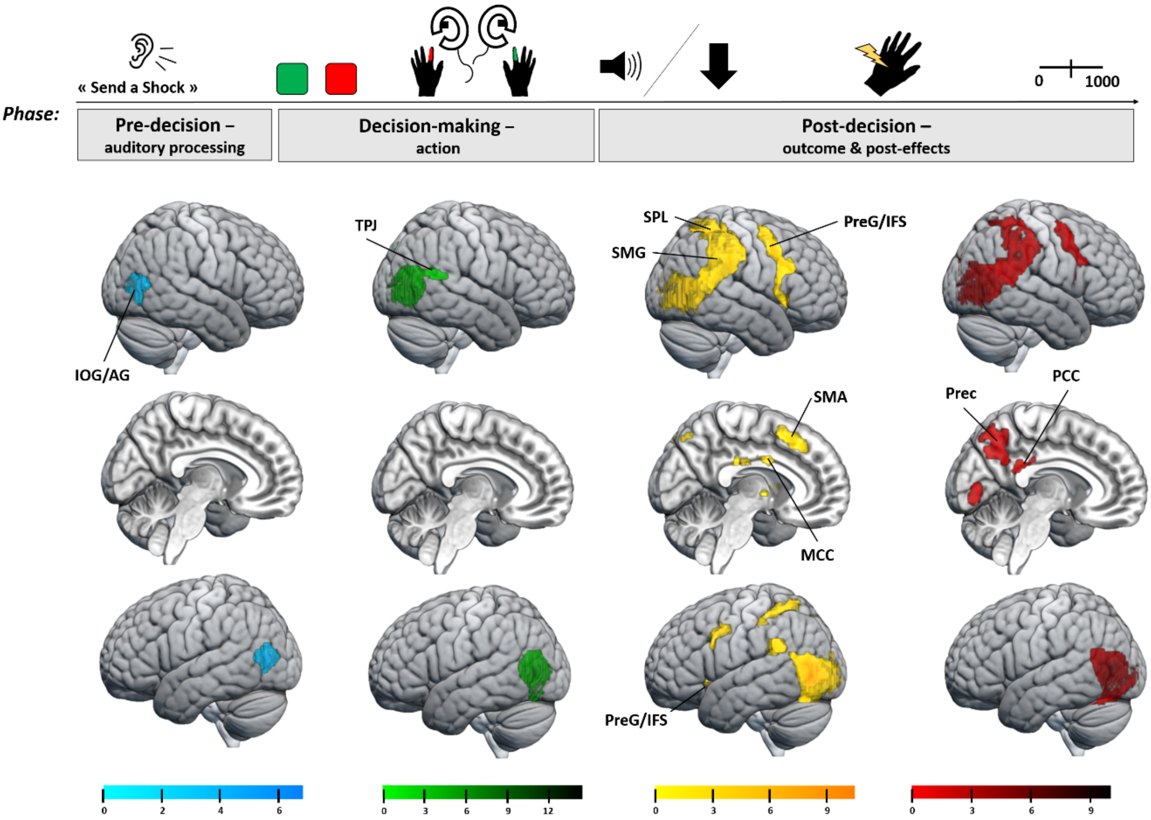
« Send a Shock »
0
1000
Phase:
Pre-decision – auditory processing
Decision-making – action
Post-decision – outcome & post-effects
TPJ
SPL
PreG/IFS
SMG
IOG/AG
SMA
Prec
PCC
MCC
PreG/IFS

Appendix D

In this exploratory analysis we compared the obedience to send a shock trial at time t that led to prosocial disobedience versus obedience to send a shock in the following trial at time t+1. We then used this contrast: [Obedience_t/Disobedience_t+1] > [Obedience_t/Obedience_t+1] on the post-decision phase for outcome and post-effects. Only 22 and 19 agents were reciprocally included in this analysis, as data of the other agents did not contain at least 5 trials by condition.

We did not find significant clusters for both epochs considering the [Obedience_t/Disobedience_t+1] > [Obedience_t/Obedience_t+1] contrast or its reverse contrast. Maybe the few trials included in this analysis did not allow to reach sufficient power, preventing us to conclude about this lack of effect.

Appendix E

In this exploratory analysis we investigated if brain activity at time t during the post-decision phase processing outcomes (during the Empathy run) could influence the behavioral decision at time t+1. For that, brain activity was modeled by adding the decision as a regressor of interest in the GLM. For this analysis to be meaningful, we only selected combination of trials where a shock was delivered at time t and where instruction to send a shock was given at time t+1. We specifically investigated if brain activity during the post-decision phase, where the victim received a shock, influenced the decision to obey or disobey the order to send a shock in the next trial. By restricting our data, we could only analyze 22 agents who had a sufficient number of relevant trials for this specific assessment (i.e., >10 trials, with at least 5 trials by decision type; Number of Obedience trials at t+1 included for each agent: [5;67]; Number of Disobedience trials at t+1 included for each agent [5;21]).

We did not find significant clusters, preventing us to conclude about the relationship between brain activity during post-decision for outcomes and subsequent behavioral choices. We are aware that this absence of effect could be due to insufficient statistical power and unbalanced number of trials included in the model between agents.

Appendix F

In this supplementary analysis we correlated the percentage of obedience to send a shock (%Anti_obe) with the 17 ROIs for all epochs. We conducted this analysis using ROIs' beta values obtained using both the ["Send a shock"/Obedience > "Send a shock"/Disobedience] and [“Send a shock”/Obedience > “Do not send a shock”/Obedience] contrasts.

By measuring ROI activity for the contrast ["Send a shock"/Obedience > "Send a shock"/Disobedience], the results show that the correlations are exactly the same as those obtained for %Pro_disob but symmetrically opposite, regardless of the epoch. More precisely, during the pre-decision phase, we observed marginal negative correlations between the antisocial obedience rate and activities in the dmPFC (r=-0.45, p_{FDR}=0.06, BF_{10}=48.88), vmPFC/ACC (r=-0.44, p_{FDR}=0.06, BF_{10}=10.90), right SMA (r=-0.42, p_{FDR}=0.06, BF_{10}=3.29) and left SMG (r=-0.42, p_{FDR}=0.06, BF_{10}=2.18). During the decision-making phase, negative correlations were found with the dmPFC (r=-0.65, p_{FDR}<0.001, BF_{10}>100), vmPFC/ACC (r=-0.58, p_{FDR}=0.004, BF_{10}>100) and bilateral SMA (left: r=-0.46, p_{FDR}=0.03, BF_{10}=7.58; right: r=-0.46, p_{FDR}=0.03, BF_{10}=9.04). These results suggest that the more agents obeyed the order to administer a shock, the more the activity in these regions was reduced. Alternatively, the lower the activity in these regions during the decision to obey compared to disobey, the more the probability to send a shock.
For the post-decision phase, we showed the involvement of the majority of ROIs, as found for the %Pro_disob, but again in the opposite direction (rs=[-0.67;-0.34], p_{FDR}s=[0.08;0.001], BF_{10}s=[0.51;>100]). Reduced involvement of this network during obedience compared to disobedience, akin to witnessing a shock versus no shock, led to greater tendencies of antisocial obedience. Alternatively, the less agents obeyed to send a shock, the more the activity in the regions associated to the post-decision phase.

Using the second contrast of interest [“Send a shock”/Obedience > “Do not send a shock”/Obedience], particularly investigating post-decision epochs, we did not fully find the same pattern of correlations than those observed for %Pro_disob. No significant correlations emerged for the pre-decision phase (all p_{FDR}s>0.1). For the decision-making phase, the same ROIs as found for %Pro_diso were negatively correlated (or marginally correlated) with the %Anti_obe: vmPFC/ACC (r=-0.56, p_{FDR}=0.004, BF_{10}>100), bilateral SMG (left: r=-0.37, p_{FDR}=0.07, BF_{10}=2.32; right: r=-0.48, p_{FDR}=0.02, BF_{10}=5.22), bilateral IOG/AG (left: r=-0.44, p_{FDR}=0.02, BF_{10}=3.83; right: r=-0.45, p_{FDR}=0.02, BF_{10}=2.33), bilateral TPJ (left: r=-0.33, p_{FDR}=0.08, BF_{10}=0.87; right: r=-0.34, p_{FDR}=0.08, BF_{10}=0.49) and right AI (r=-0.38, p_{FDR}=0.06, BF_{10}=1.36). It suggests that the more agents obeyed the order to administer a shock, the lower the activity in these regions during the decision-making phase was. Alternatively, the higher the activity in these regions was, the less the probability to obey to send a shock was.

For the post-decision considering the outcome period, the same ROIs than those observed for %Pro_disob were found negatively correlated with %Anti_obe, with the

addition of a marginal correlation with the left SPL: bilateral IOG/AG (left: r=-0.58, p_{FDR}=0.001, BF_{10}>100; right: r=-0.49, p_{FDR}=0.007, BF_{10}>100), bilateral TPJ (left: r=-0.49, p_{FDR}=0.007, BF_{10}=5.06; right: r=-0.37, p_{FDR}=0.05, BF_{10}=2.75), bilateral AI (left: r=-0.32, p_{FDR}=0.1, BF_{10}=2.81; right: r=-0.42, p_{FDR}=0.03, BF_{10}=5.80), Prec/PCC (r=-0.38, p_{FDR}=0.05, BF_{10}=5.16) and left SPL (r=-0.32, p_{FDR}=0.1, BF_{10}=0.98). These results indicate that the more frequently agents obeyed the experimenter's order to send a shock, the less the brain regions associated with the outcome period were activated. Alternatively, the higher the activity in these regions during the outcome period was, the less the probability to obey to send a shock was.

Finally, for the post-effects period, almost all ROIs were found significantly (or marginally) negatively correlated with the %Anti_obe (rs=[-0.60;-0.30], p_{FDR}s=[0.08;0.001], BF_{10}s=[0.58;>100]), with the exception of the right SPL, revealing again mirror results compared to %Pro_disob. The more frequently agents followed the orders to send a shock, the lower the brain activity during this post-effect period. Alternatively, the higher the brain activity during this post-effect period was, the less the agents decided to send a shock.

Appendix G

Individual plots (left side) and group plots (right side) showing the decision to prosocially disobey across time. Curves represent the cumulative %Pro_disob (up to 80, as the instruction to send a shock was given 80 times) over the 120 trials for both Agency (top) and Empathy (bottom) runs. Overall, these plots revealed three different profiles: 1) participants showing no prosocial disobedience (flat curve, for example, participant no. 20 highlighted in blue), 2) participants exhibiting a strong altruistic profile, almost consistently disobeying throughout the task (linearly increasing curve, for example, participant no. 46 highlighted in red), and 3) participants showing more trial-by-trial choices, disobeying on certain trials (jagged curve, for example, participant no. 5 highlighted in purple). Some plots also show plateau where participants took time before starting to disobey or stopped disobeying towards the end of the task (curves with plateau, for example, participants no. 7 in Agency and 37 in Empathy highlighted in yellow). Below, you can view a zoomed-in figure of the agents provided as examples.
It is important to note that participants were generally consistent across the two runs in their decision-making strategy.

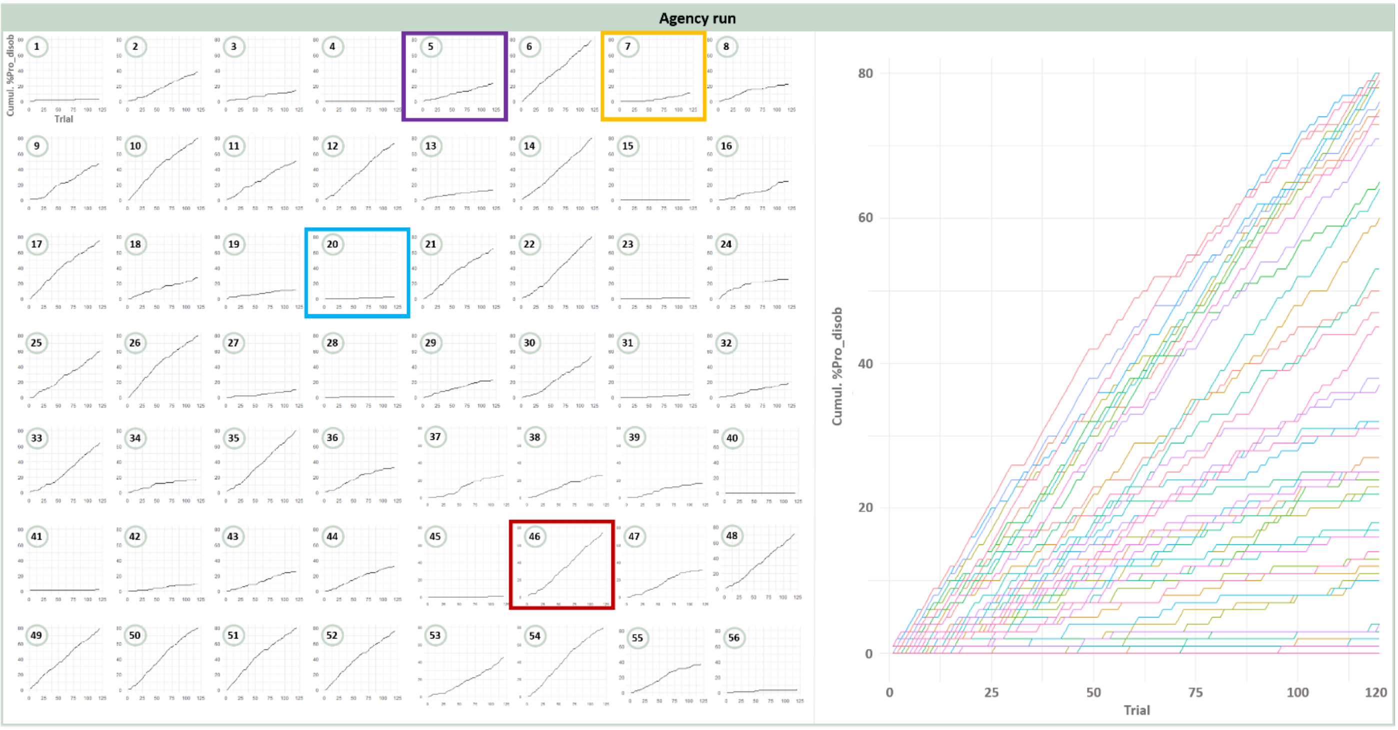

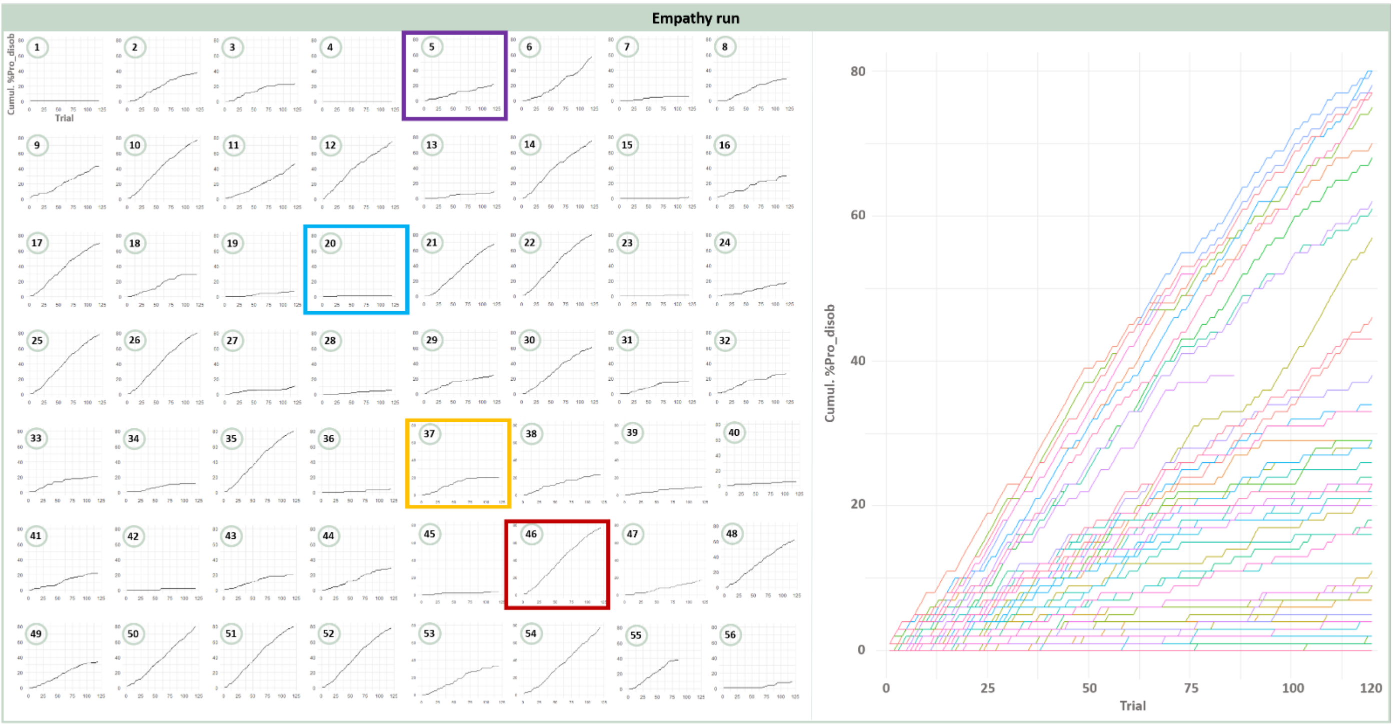
Empathy run
Cumul. %Pro_disob
Trial

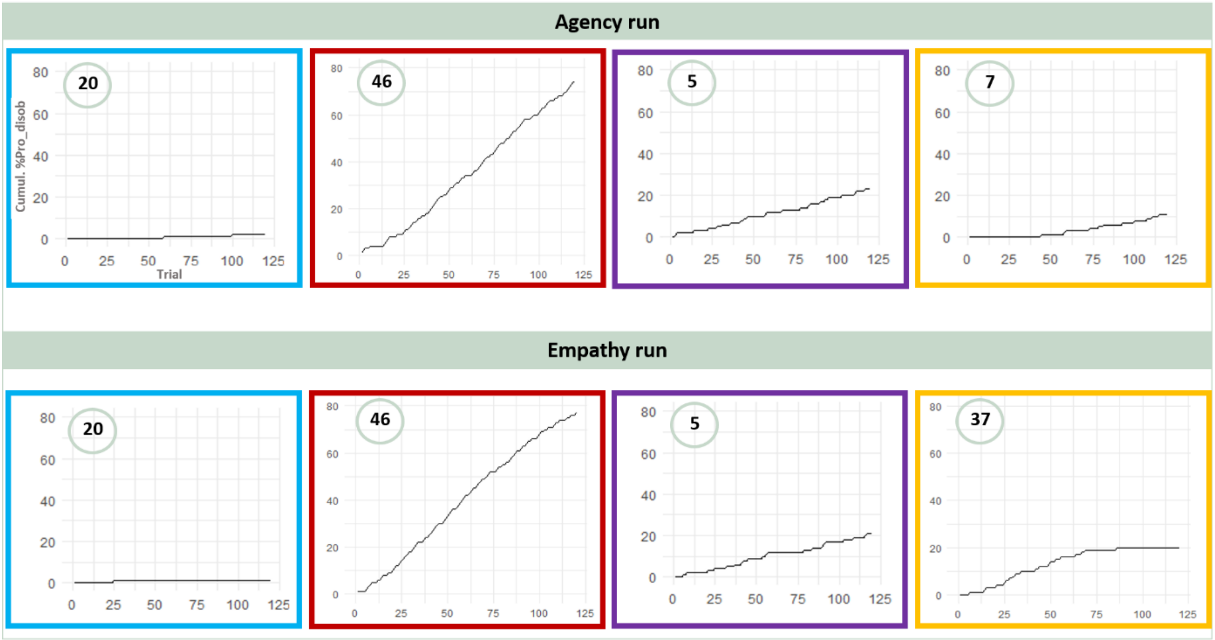
Agency run
Cumul. %Pro_disob
Trial
Empathy run

Appendix H

Response profile for each agent and each run (Agency, Empathy), expressed in percentage (%) for each four conditions ("Send a shock"/Obedience, "Send a shock"/Disobedience, "Do not send a shock"/Obedience, "Do not send a shock"/Disobedience). Agents showing a high prosocial behavior are highlighted in blue, those showing a high antisocial behavior are highlighted in red, and agents who rarely or neither disobeyed whatever the received order are highlighted in yellow. The other agents presented a more heterogeneous profile with a balance rate of obedience and disobedience.

Agent	Agency run				Empathy run			
	**"Send a shock"/ Obedience**	**"Send a shock"/ Disobedience**	**"Do not send a shock"/ Obedience**	**"Do not send a shock"/ Disobedience**	**"Send a shock"/ Obedience**	**"Send a shock"/ Disobedience**	**"Do not send a shock"/ Obedience**	**"Do not send a shock"/ Disobedience**
AVX8	64.17%	2.50	32.50	0.83	65.83	0.83	32.50	0.83
AP2R	35.00	31.67	33.33	0.00	35.00	31.67	33.33	0.00
AFL0	55.00	11.67	30.83	2.50	47.50	19.17	30.83	2.50
BGJ1	66.67	0.00	0.83	32.50	66.67	0.00	0.00	33.33
BF2P	27.50	39.17	29.17	4.17	30.83	35.83	30.83	2.50
BMM0	0.00	66.67	32.50	0.83	2.50	64.17	32.50	0.83
BZ1Y	25.00	41.67	30.83	2.50	28.33	38.33	33.33	0.00
CVB9	5.83	60.83	30.83	2.50	4.17	62.50	28.33	5.00
D1NG	4.17	62.50	31.67	1.67	8.33	58.33	33.33	0.00
D7FY	44.17	22.50	23.33	10.00	42.50	24.17	29.17	4.17
EII9	56.67	10.00	32.50	0.83	60.83	5.83	32.50	0.83
FYA7	65.00	1.67	0.00	33.33	65.83	0.83	0.00	33.33
GA2F	16.67	50.00	33.33	0.00	1.67	65.00	33.33	0.00
GB2L	0.00	66.67	33.33	0.00	0.00	66.67	33.33	0.00
GCV5	58.33	8.33	28.33	5.00	57.50	9.17	33.33	0.00
H3LI	65.83	0.83	32.50	0.83	62.50	4.17	30.00	3.33
HT5X	47.50	19.17	11.67	21.67	49.17	17.50	13.33	20.00
HH6S	1.67	65.00	33.33	0.00	19.17	47.50	31.67	1.67
HJS8	57.50	9.17	31.67	1.67	62.50	4.17	32.50	0.83
I0VB	48.33	18.33	31.67	1.67	43.33	23.33	33.33	0.00
JHJ7	55.83	10.83	33.33	0.00	59.17	7.50	30.83	2.50
JK8Q	0.00	66.67	33.33	0.00	4.17	62.50	33.33	0.00
KOU0	66.67	0.00	5.00	28.33	65.00	1.67	5.00	28.33
KW1Z	46.67	20.00	22.50	10.83	42.50	24.17	19.17	14.17
K4MY	12.50	54.17	33.33	0.00	10.00	56.67	32.50	0.83
LZ4C	0.00	66.67	33.33	0.00	0.00	66.67	33.33	0.00
MN5V	65.83	0.83	31.67	1.67	65.83	0.83	32.50	0.83
MRO8	45.83	20.83	30.83	2.50	51.67	15.00	30.00	3.33
NP2D	48.33	18.33	30.83	2.50	46.67	20.00	31.67	1.67
OZY4	22.50	44.17	24.17	9.17	15.83	50.83	26.67	6.67
OLQ6	63.33	3.33	30.00	3.33	53.33	13.33	33.33	0.00
OPQ8	51.67	15.00	20.83	12.50	45.00	21.67	20.83	12.50

PG1D	13.33	53.33	31.67	1.67	49.17	17.50	31.67	1.67
P2SM	52.50	14.17	32.50	0.83	56.67	10.00	33.33	0.00
PR8O	0.00	66.67	33.33	0.00	0.00	66.67	33.33	0.00
Q4TU	--	--	--	--	63.33	3.33	30.00	3.33
QV7Q	40.00	26.67	16.67	16.67	48.33	18.33	15.00	18.33
Q8ML	65.00	1.67	0.00	33.33	65.00	1.67	0.00	33.33
R6XX	58.33	8.33	25.83	7.50	50.00	16.67	28.33	5.00
RGH8	45.83	20.83	21.67	11.67	42.50	24.17	22.50	10.83
S3HB	40.00	26.67	31.67	1.67	38.33	28.33	31.67	1.67
S7VN	0.83	65.83	33.33	0.00	--	--	--	--
SG8Z	0.00	66.67	33.33	0.00	0.00	66.67	32.50	0.83
TFT4	0.00	66.67	33.33	0.00	0.00	66.67	33.33	0.00
TOI5	3.33	63.33	33.33	0.00	1.67	65.00	33.33	0.00
TD6Q	66.67	0.00	0.83	32.50	62.50	4.17	0.83	32.50
T9CV	7.50	59.17	32.50	0.83	15.00	51.67	33.33	0.00
UUI0	63.33	3.33	30.83	2.50	59.17	7.50	23.33	10.00
UK4K	35.83	30.83	15.00	18.33	27.91	44.19	15.12	12.79
U5RT	45.83	20.83	31.67	1.67	50.00	16.67	30.83	2.50
UCJ7	45.83	20.83	32.50	0.83	47.50	19.17	31.67	1.67
VBB1	53.33	13.33	25.00	8.33	59.17	7.50	23.33	10.00
V2SA	65.83	0.83	30.83	2.50	63.33	3.33	31.67	1.67
VRT5	5.00	61.67	32.50	0.83	2.50	64.17	33.33	0.00
VG8H	40.83	25.83	28.33	5.00	52.50	14.17	30.00	3.33
WZY2	29.17	37.50	20.00	13.33	39.17	27.50	17.50	15.83
WXP3	0.83	65.83	33.33	0.00	2.50	64.17	33.33	0.00

Appendix I

Results obtained using the [“Send a shock”/Obedience > “Send a shock”/Disobedience] contrast for the post-decision phase.

Outcome period: As illustrated by the figure below, results revealed several significant clusters including the bilateral IOG/AG but also the right SMG extending to the TPJ and the Superior Parietal Lobule (SPL), the left SMG, the left SPL, the right Precentral Gyrus (PreG) extending to the Precentral Sulcus (PreS) and the Inferior Frontal Sulcus (IFS), and the SMA (see the table below). By adding the exclusive mask, we found a cluster in the left TPJ extending to SMG ([-61 -43 24], Z = 5.25, cluster size = 266, p=0.005). No significant cluster was found for the reverse contrast. The [“Send a shock”/Obedience > “Do not send a shock”/Obedience] contrast revealed similar results, with no significant cluster on the reverse contrast.

Post-effects period: Results revealed four significant clusters in bilateral IOG/AG extending to TPJ, in right SMG and in right PreG extending to PreS (figure and table below). These results were comparable with those obtained for the [“Send a shock”/Obedience > “Do not send a shock”/Obedience] contrast (Appendix B). By adding the exclusive mask on the [“Send a shock”/Obedience > “Send a shock”/Disobedience] contrast we found a cluster on the left hemisphere including the AG, TPJ and SMG ([-52 -56 15], Z = 4.96, cluster size = 609, p=0.005). No significant cluster was found for the reverse contrasts.
No significant cluster was found on this contrast when interacting with the Run factor, suggesting a similar modulation of the networks in Agency and Empathy runs.

Anatomical location	MNI coordinates (in mm)			Z score	Cluster size (number of voxels)
[“Send a shock”/Obedience > ”Send a shock”/Disobedience]	x	y	z		
Post-decision phase for outcome					
Left Inferior Occipital Gyrus / Angular Gyrus	-47	-74	6	6.98	1572
Right Inferior Occipital Gyrus / Angular Gyrus	50	-70	3	5.94	1368
Left Supramarginal Gyrus	-61	-43	24	4.46	126
Left Superior Parietal Lobule	-38	-47	63	4.72	95
Right Supramarginal Gyrus / Temporo-Parietal Junction / Superior Parietal Lobule	66	-22	45	5.73	964
Right Precentral Gyrus / Precentral Sulcus / Inferior Frontal Sulcus	43	0	60	4.13	445
Supplementary Motor Area	3	23	51	3.94	98
Post-decision phase for post-effects					

Left Inferior Occipital Gyrus / Angular Gyrus / Temporo-Parietal Junction	-47	-76	6	6.60	1344
Right Inferior Occipital Gyrus / Angular Gyrus / Temporo-Parietal Junction	50	-72	-4	6.44	1239
Right Supramarginal Gyrus	68	-27	39	4.89	296
Right Precentral Gyrus / Precentral Sulcus	54	7	42	4.17	265

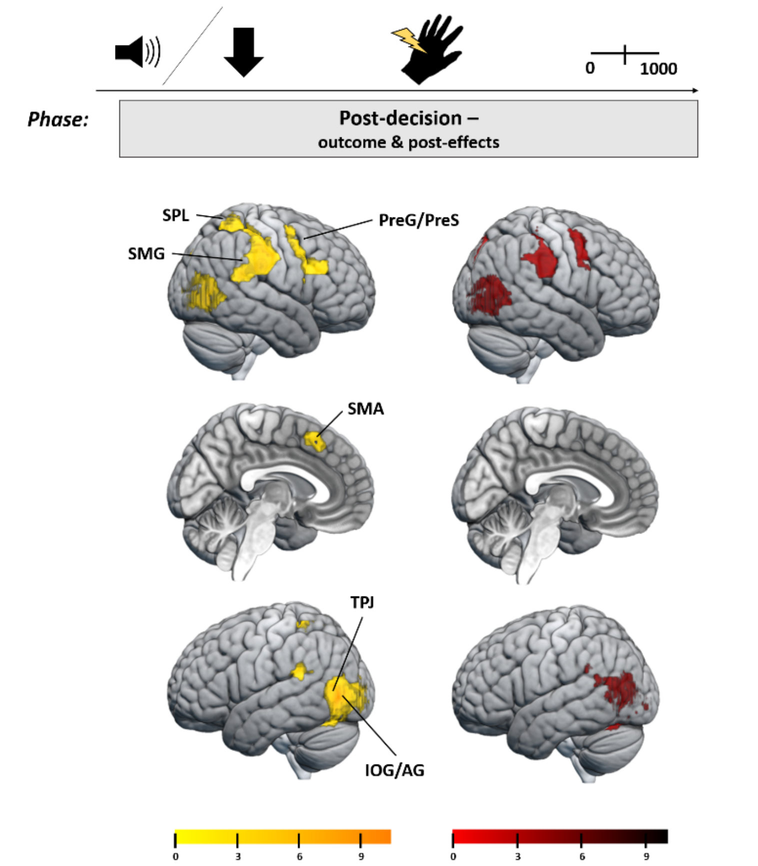

Appendix J

Correlations between the 17 ROIs activity and the %Pro-disob using the ["Send a shock"/Obedience > "Send a shock"/Disobedience] contrast and the ["Send a shock"/Obedience > "Do not send a shock"/Obedience]. Statistics for all correlations are given in the following table. Plots represent correlations for each ROI, in each epoch and for each contrast. The following paragraphs complement the results described in the main manuscript.

Post-decision phases using the ["Send a shock"/Obedience > "Send a shock"/Disobedience] contrast: For the outcome period, there was a positive correlation between the majority of the ROIs and the %Pro_disob, corroborated by the Bayesian approach for all but bilateral TPJ and right SMG ROIs: vmPFC (r=0.45, p_{FDR}=0.03, BF_{10}=5.35), bilateral IOG/AG (left: r=0.41, p_{FDR}=0.04, BF_{10}=12.65; right: r=0.50, p_{FDR}=0.02, BF_{10}=176.97), bilateral TPJ (left: r=0.39, p_{FDR}=0.04, BF_{10}=0.54; right: r=0.45, p_{FDR}=0.03, BF_{10}=0.67), bilateral SPL (left: r=0.58, p_{FDR}=0.005, BF_{10}=161.29; right: r=0.41, p_{FDR}=0.04, BF_{10}=9.29), right AI (r=0.42, p_{FDR}=0.04, BF_{10}=0.40), right SMG (r=0.37, p_{FDR}=0.05, BF_{10}=0.51) and right PreG (r=0.34, p_{FDR}=0.08, BF_{10}=3.64). The Bayesian approach also indicated moderate evidence toward H1 for Prec/PCC (BF_{10}=4.58). For the post-effects period, several positive correlations also emerged, including dmPFC (r=0.67, p_{FDR}=0.001, BF_{10}=2674.36), vmPFC/ACC (r=0.51, p_{FDR}=0.01, BF_{10}=106.21), bilateral IOG/AG (left: r=0.48, p_{FDR}=0.02, BF_{10}=2.81; right: r=0.45, p_{FDR}=0.03, BF_{10}=6.39), left SMG (r=0.42, p_{FDR}=0.05, BF_{10}=4.81), bilateral SMA (left: r=0.54, p_{FDR}=0.01, BF_{10}=14.83; right: r=0.58, p_{FDR}=0.007, BF_{10}=38.54) and right PreG (r=0.50, p_{FDR}=0.02, BF_{10}=33.56).

Pre-decision and decision-making phases using the ["Send a shock"/Obedience > "Do not send a shock"/Obedience] contrast: For the pre-decision phase, we did not find significant correlations. For the decision-phase, a positive correlation was found between %Pro-disob and four ROIs: bilateral IOG/AG (left: r=0.44, p_{FDR}=0.02, BF_{10}=3.83; right: r=0.45, p_{FDR}=0.02, BF_{10}=2.33), right SMG (r=0.48, p_{FDR}=0.02, BF_{10}=5.22) and vmPFC/ACC (r=0.56, p_{FDR}=0.004, BF_{10}=359.26). We also found marginal correlations with left SMG (r=0.37, p_{FDR}=0.07, BF_{10}=2.32), bilateral TPJ (left: r=0.33, p_{FDR}=0.08, BF_{10}=0.87; right: r=0.34, p_{FDR}=0.08, BF_{10}=0.49) and right AI (r=0.38, p_{FDR}=0.06, BF_{10}=1.36).

ROI	Pre-decision phase<br>Auditory processing	Decision-making phase<br>Action	Post-decision phase<br>Outcome	Post-decision phase<br>Post-effects
**["Send a shock"/Obedience > "Send a shock"/Disobedience]**				
**Left IOG/AG**	r=-0.04, p_{FDR}=0.81, BF_{10}=0.27	r=0.14, p_{FDR}=0.56, BF_{10}=0.23	r=0.41, p_{FDR}=0.04, BF_{10}=12.65	r=0.48, p_{FDR}=0.02, BF_{10}=2.81
**Right IOG/AG**	r=-0.06, p_{FDR}=0.80, BF_{10}=0.25	r=0.26, p_{FDR}=0.31, BF_{10}=0.24	r=0.50, p_{FDR}=0.02, BF_{10}=176.97	r=0.45, p_{FDR}=0.03, BF_{10}=6.39
**Left TPJ**	r=0.14, p_{FDR}=0.66, BF_{10}=0.23	r=0.07, p_{FDR}=0.78, BF_{10}=0.22	r=0.39, p_{FDR}=0.04, BF_{10}=0.54	r=0.09, p_{FDR}=0.65, BF_{10}=0.26
**Right TPJ**	r=-0.15, p_{FDR}=0.66, BF_{10}=0.41	r=0.16, p_{FDR}=0.56, BF_{10}=0.22	r=0.45, p_{FDR}=0.03, BF_{10}=0.67	r=0.23, p_{FDR}=0.30, BF_{10}=0.25
**Left AI**	r=0.36, p_{FDR}=0.14, BF_{10}=0.31	r=0.16, p_{FDR}=0.56, BF_{10}=0.29	r=0.31, p_{FDR}=0.11, BF_{10}=0.95	r=0.11, p_{FDR}=0.65, BF_{10}=0.26
**Right AI**	r=0.26, p_{FDR}=0.31, BF_{10}=0.39	r=0.22, p_{FDR}=0.41, BF_{10}=0.27	r=0.42, p_{FDR}=0.04, BF_{10}=0.40	r=0.35, p_{FDR}=0.10, BF_{10}=0.69

**Left PreG**	r=0.24, p_{FDR}=0.35, BF_{10}=0.48	r=0.05, p_{FDR}=0.82, BF_{10}=0.26	r=0.16, p_{FDR}=0.39, BF_{10}=0.27	r=0.30, p_{FDR}=0.16, BF_{10}=0.49
**Right PreG**	r=0.26, p_{FDR}=0.31, BF_{10}=0.84	r=0.26, p_{FDR}=0.31, BF_{10}=0.42	r=0.34, p_{FDR}=0.08, BF_{10}=3.64	r=0.50, p_{FDR}=0.02, BF_{10}=33.56
**Left SPL**	r=0.09, p_{FDR}=0.79, BF_{10}=0.24	r=0.28, p_{FDR}=0.31, BF_{10}=0.27	r=0.58, p_{FDR}=0.005, BF_{10}=161.29	r=0.35, p_{FDR}=0.10, BF_{10}=2.47
**Right SPL**	r=0.13, p_{FDR}=0.66, BF_{10}=0.22	r=0.08, p_{FDR}=0.78, BF_{10}=0.22	r=0.41, p_{FDR}=0.04, BF_{10}=9.29	r=0.18, p_{FDR}=0.43, BF_{10}=0.43
**Left SMG**	r=0.42, p_{FDR}=0.06, BF_{10}=2.18	r=0.37, p_{FDR}=0.12, BF_{10}=0.86	r=0.29, p_{FDR}=0.12, BF_{10}=2.72	r=0.42, p_{FDR}=0.05, BF_{10}=4.80
**Right SMG**	r=-0.07, p_{FDR}=0.80, BF_{10}=0.23	r=0.14, p_{FDR}=0.56, BF_{10}=0.23	r=0.37, p_{FDR}=0.05, BF_{10}=0.51	r=0.15, p_{FDR}=0.52, BF_{10}=0.48
**Left SMA**	r=0.29, p_{FDR}=0.27, BF_{10}=2.41	r=0.46, p_{FDR}=0.03, BF_{10}=7.57	r=0.27, p_{FDR}=0.14, BF_{10}=0.53	r=0.54, p_{FDR}=0.01, BF_{10}=14.83
**Right SMA**	r=0.42, p_{FDR}=0.06, BF_{10}=3.29	r=0.46, p_{FDR}=0.03, BF_{10}=9.04	r=0.24, p_{FDR}=0.19, BF_{10}=0.50	r=0.58, p_{FDR}=0.007, BF_{10}=38.54
**Prec/PCC**	r=0.07, p_{FDR}=0.80, BF_{10}=0.29	r=0.01, p_{FDR}=0.97, BF_{10}=0.40	r=0.28, p_{FDR}=0.14, BF_{10}=4.58	r=0.07, p_{FDR}=0.70, BF_{10}=0.23
**dmPFC**	r=0.45, p_{FDR}=0.06, BF_{10}=48.89	r=0.65, p_{FDR}=0.001, BF_{10}=712.31	r=-0.0.4, p_{FDR}=0.83, BF_{10}=0.21	r=0.67, p_{FDR}=0.001, BF_{10}>1000
**vmPFC/ACC**	r=0.44, p_{FDR}=0.06, BF_{10}=10.89	r=0.58, p_{FDR}=0.004, BF_{10}=252.91	r=0.45, p_{FDR}=0.03, BF_{10}=5.35	r=0.51, p_{FDR}=0.01, BF_{10}=106.21
**[“Send a shock”/Obedience > “Do not send a shock”/Obedience]**				
**Left IOG/AG**	r=0.19, p_{FDR}=0.38, BF_{10}=0.26	r=0.44, p_{FDR}=0.02, BF_{10}=3.83	r=0.56, p_{FDR}=0.002, BF_{10}=101.89	r=0.58, p_{FDR}=0.001, BF_{10}=43.92
**Right IOG/AG**	r=0.27, p_{FDR}=0.22, BF_{10}=0.26	r=0.45, p_{FDR}=0.02, BF_{10}=2.33	r=0.49, p_{FDR}=0.01, BF_{10}=157.40	r=0.49, p_{FDR}=0.004, BF_{10}=153.36
**Left TPJ**	r=0.45, p_{FDR}=0.07, BF_{10}=0.34	r=0.33, p_{FDR}=0.08, BF_{10}=0.87	r=0.40, p_{FDR}=0.04, BF_{10}=3.07	r=0.29, p_{FDR}=0.08, BF_{10}=1.51
**Right TPJ**	r=0.21, p_{FDR}=0.34, BF_{10}=0.21	r=0.34, p_{FDR}=0.08, BF_{10}=0.49	r=0.37, p_{FDR}=0.06, BF_{10}=3.08	r=0.42, p_{FDR}=0.01, BF_{10}=8.98
**Left AI**	r=0.34, p_{FDR}=0.16, BF_{10}=0.30	r=0.28, p_{FDR}=0.15, BF_{10}=0.24	r=0.34, p_{FDR}=0.08, BF_{10}=2.91	r=0.50, p_{FDR}=0.004, BF_{10}=10.51
**Right AI**	r=0.34, p_{FDR}=0.16, BF_{10}=0.63	r=0.38, p_{FDR}=0.06, BF_{10}=1.36	r=0.34, p_{FDR}=0.08, BF_{10}=2.70	r=0.43, p_{FDR}=0.01, BF_{10}=7.32
**Left PreG**	r=0.13, p_{FDR}=0.58, BF_{10}=0.21	r=0.005, p_{FDR}=0.97, BF_{10}=0.20	r=0.08, p_{FDR}=0.64, BF_{10}=0.20	r=0.26, p_{FDR}=0.11, BF_{10}=0.56
**Right PreG**	r=0.07, p_{FDR}=0.65, BF_{10}=0.21	r=0.25, p_{FDR}=0.16, BF_{10}=0.57	r=0.20, p_{FDR}=0.23, BF_{10}=0.31	r=0.40, p_{FDR}=0.02, BF_{10}=19.18
**Left SPL**	r=0.11, p_{FDR}=0.58, BF_{10}=0.20	r=0.08, p_{FDR}=0.66, BF_{10}=0.21	r=0.24, p_{FDR}=0.16, BF_{10}=0.53	r=0.32, p_{FDR}=0.06, BF_{10}=1.32
**Right SPL**	r=0.11, p_{FDR}=0.58, BF_{10}=0.20	r=0.12, p_{FDR}=0.52, BF_{10}=0.21	r=0.21, p_{FDR}=0.22, BF_{10}=0.44	r=0.29, p_{FDR}=0.08, BF_{10}=1.98
**Left SMG**	r=0.30, p_{FDR}=0.20, BF_{10}=0.44	r=0.37, p_{FDR}=0.07, BF_{10}=2.32	r=0.27, p_{FDR}=0.14, BF_{10}=1.49	r=0.48, p_{FDR}=0.005, BF_{10}=68.43
**Right SMG**	r=0.22, p_{FDR}=0.34, BF_{10}=0.43	r=0.48, p_{FDR}=0.02, BF_{10}=5.22	r=0.26, p_{FDR}=0.14, BF_{10}=0.71	r=0.45, p_{FDR}=0.01, BF_{10}=0.51
**Left SMA**	r=0.29, p_{FDR}=0.20, BF_{10}=0.43	r=0.25, p_{FDR}=0.16, BF_{10}=0.46	r=0.28, p_{FDR}=0.14, BF_{10}=0.80	r=0.43, p_{FDR}=0.01, BF_{10}=15.03
**Right SMA**	r=0.26, p_{FDR}=0.24, BF_{10}=0.44	r=0.23, p_{FDR}=0.20, BF_{10}=1.00	r=0.26, p_{FDR}=0.14, BF_{10}=0.79	r=0.46, p_{FDR}=0.01, BF_{10}=51.51
**Prec/PCC**	r=0.08, p_{FDR}=0.65, BF_{10}=0.20	r=0.27, p_{FDR}=0.15, BF_{10}=0.23	r=0.39, p_{FDR}=0.04, BF_{10}=5.93	r=0.52, p_{FDR}=0.003, BF_{10}=6.38
**dmPFC**	r=0.18, p_{FDR}=0.39, BF_{10}=0.65	r=0.30, p_{FDR}=0.12, BF_{10}=5.06	r=0.19, p_{FDR}=0.26, BF_{10}=0.50	r=0.55, p_{FDR}=0.002, BF_{10}>1000
**vmPFC/ACC**	r=0.35, p_{FDR}=0.16, BF_{10}=1.59	r=0.56, p_{FDR}=0.004, BF_{10}=359.26	r=0.26, p_{FDR}=0.14, BF_{10}=0.59	r=0.61, p_{FDR}<0.001, BF_{10}=142.79

[“Send a shock”/Obedience > “Send a shock”/Disobedience]

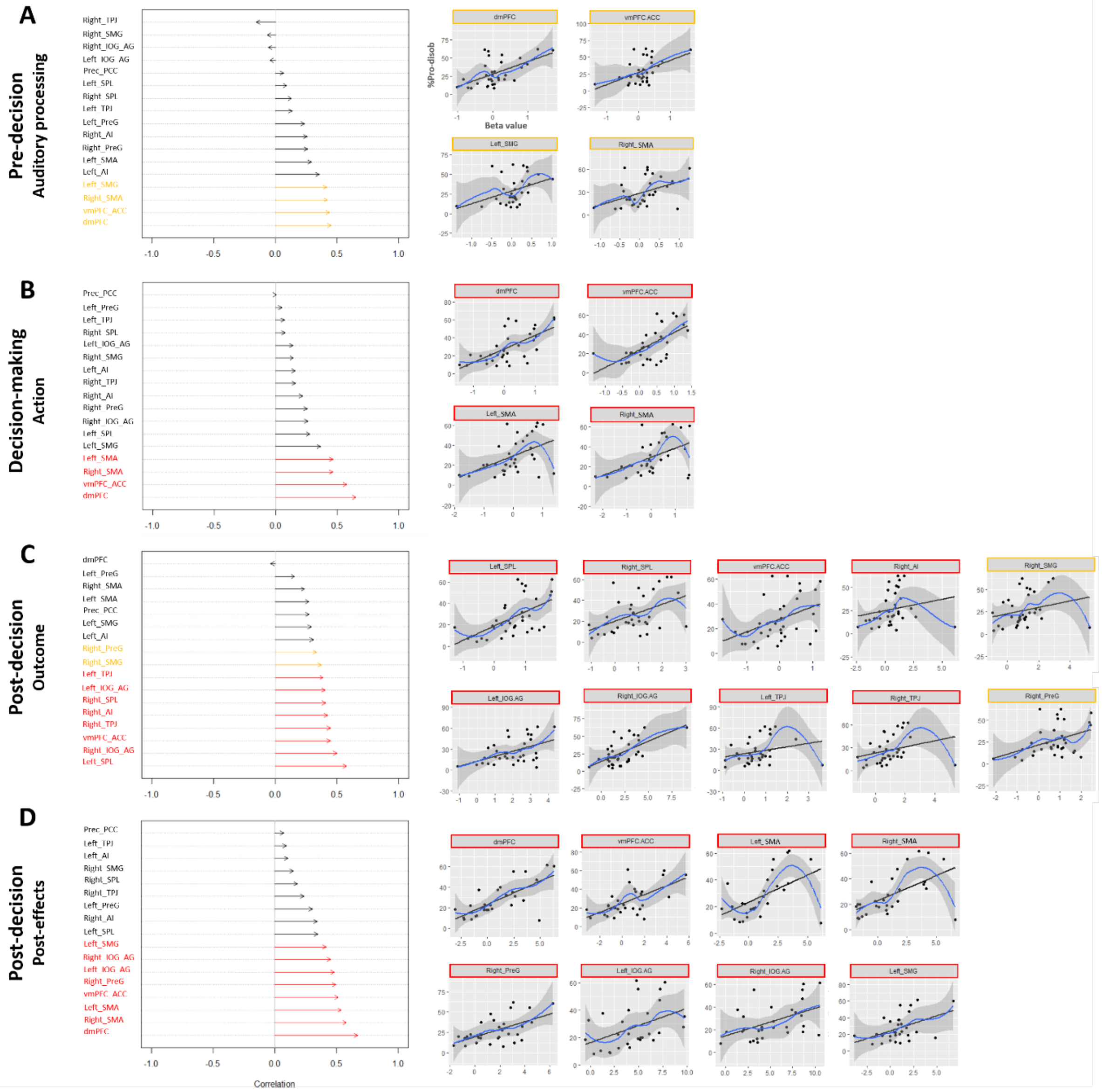

[“Send a shock”/Obedience > “Do not send a shock”/Obedience]

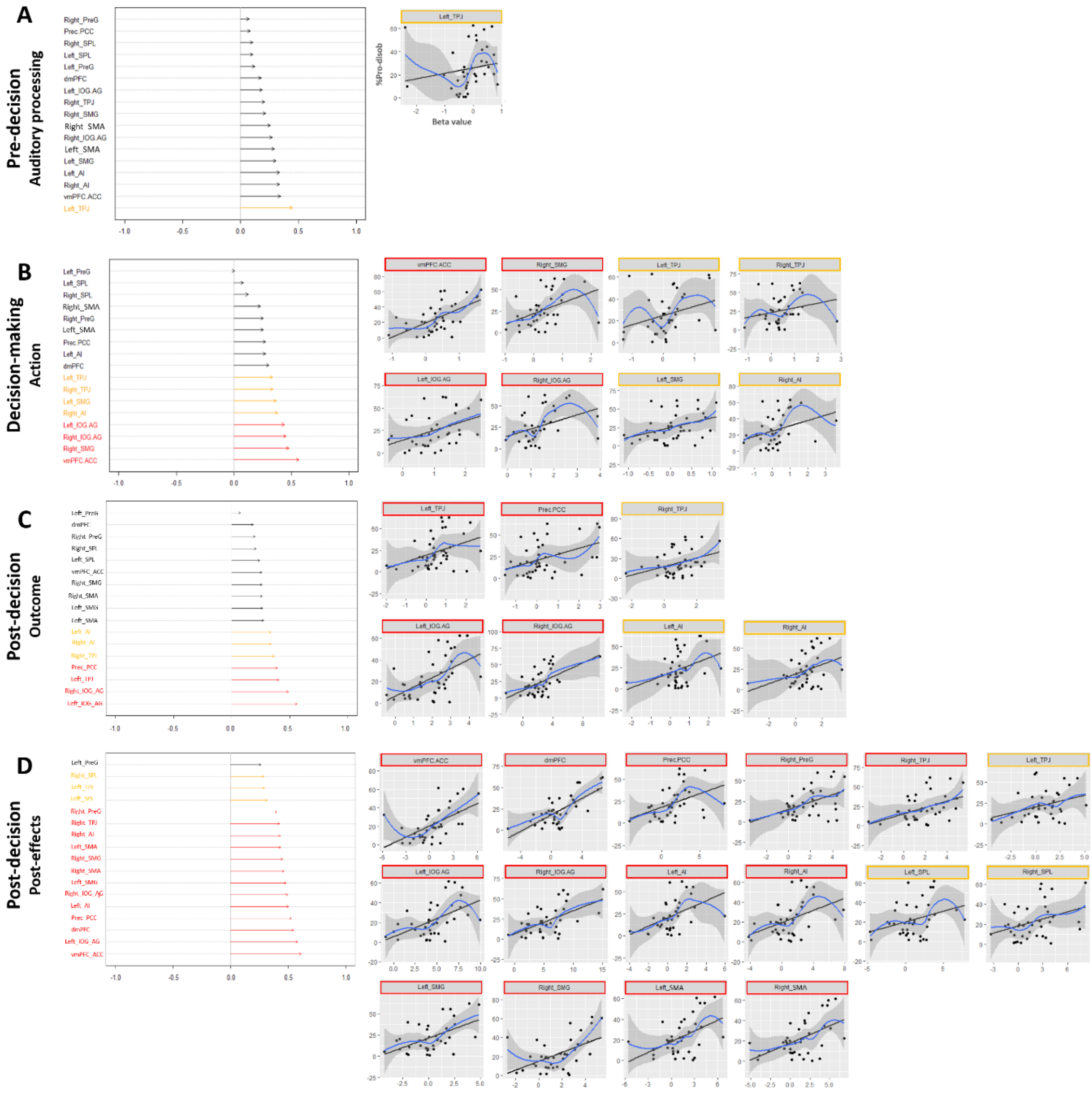

Appendix K

The unequal number of disobedience trials among participants lead to substantial inter-individual variabilities and variable signal-to-noise ratios that could influence the imaging results in correlation analyses. To control for that, we conducted a supplementary analysis by randomly selecting a fixed number of obedience and disobedience trials for all participants to measure brain activity for each ROI. We chose to fix the trial number at 10 to retain a reasonable number of participants (between 22 and 27 depending on the decision phase analyzed). The 10 "Obedience to send a shock" trials and the 10 "Disobedience to send a shock" trials were randomly selected for each participant to create the "Send a shock"/Obedience > "Send a shock"/Disobedience contrast. The correlations between each ROI activity and %Pro_disob was conducted similarly as analyses reported in the main manuscript.

The results from this supplementary analysis corroborated those from our primary analyses for both the pre-decision and decision-making phases. No significant correlations were found between %Pro_disob and the ROIs during the pre-decision phase. During the decision-making phase, we observed a positive correlation between %Pro_disob and six ROIs, including again the dmPFC (r=0.54, p_{FDR}=0.02, BF_{10}=52.95), vmPFC/ACC (r=0.48, p_{FDR}=0.04, BF_{10}=6.63), bilateral SMA (left: r=0.53, p_{FDR}=0.02, BF_{10}=10.44; right: r=0.47, p_{FDR}=0.04, BF_{10}=6.39), with the addition of the left SMG (r=0.57, p_{FDR}=0.02, BF_{10}=66.12) and the right PreG (r=0.47, p_{FDR}=0.04, BF_{10}=8.03). However, we did not replicate the results for the post-decision phases, as no correlations remained significant (all p_{FDR}s>0.3).

However, this decision of selecting 10 trials for each condition, while retaining more participants, also resulted in the loss of numerous trials, thereby diminishing overall statistical power. It also required that participants had a sufficiently large and equivalent number of trials in both conditions. This approach necessitated excluding highly altruistic agents (i.e., with many disobedience trials and fewer obedience trials) as well as highly obedient agents (i.e., with many obedience trials and fewer disobedience trials). Yet, inter-individual variability is crucial to explore in studies investigating social influences on behavior, particularly in correlational analyses.

Appendix L

Supplementary analyses to test the hypothesis of a modulation of attention according to the decision type. In fact, attention variability could influence the decision to obey or disobey. However, we don't have evidence from previous studies on obedience (Caspar et al., 2020, 2021) and disobedience (Caspar et al., 2022) suggesting an attentional effect.
*In the present study, for the decision-making phase we looked at the detection times (DTs) over time (i.e., 120 trials) for both Agency and Empathy runs, comparing obedience and disobedience to send a shock conditions. Indeed, DT (or reaction time) is one of the behavioral markers that captures attentional processes and the mental effort associated with a task (Smith et al., 2004; Yamashita et al., 2021), and is commonly used in dual-choice decision-making models (Ratcliff & McKoon, 2008; Ratcliff & Rouder, 1998). We investigated the Time x Decision type effect using a regression model (Used linear model: log(DTs) ~ Trial * Decision type + (1|(Agent)).*
Agents completed agency and empathy tasks concurrent with their decisions to obey or disobey, partially allowing us to ensure that attention remained consistent over time (particularly with regard to the SoA task which require a great attention to action-outcome intervals). Then for the post-decision phase, we investigated SoA scores, and specifically if the number of outliers varied with the decision to obey or disobey. Indeed, we quantified the number of outliers for each participant using the Interquartile Range (IQR) method with a threshold of 1.5xIQR. We then assessed whether there was a difference in the number of outliers between the Obedience and Disobedience conditions: Aov1, number_outliers ~ Obedience.

Decision-making phase results (i.e., DTs): The regression analysis showed a main effect of Trial on DTs (Agency: est.=-0.002, t=-3.66, p<0.001; Empathy: est.=-0.002, t=-4.83, p<0.001), without interaction with the decision type (ps>0.10). This effect of Trial was not surprising, as number of evidences in the literature report a modulation of performance across time (Thomson et al., 2015). However, this fluctuation is not modulated by the decision type. Obtained plots are given below.

Post-decision making phases results (i.e., number of outliers in SoA scores): Results showed no effect on both the Z-score (F(1,55)=0.19, p=0.66), and the Error-score (F(1,55)=0.06, p=0.80). There was no increase in the number of outliers for the SoA scores between the obedience and disobedience conditions, suggesting that attention level did not depend on the decision-making-type.

Agency run

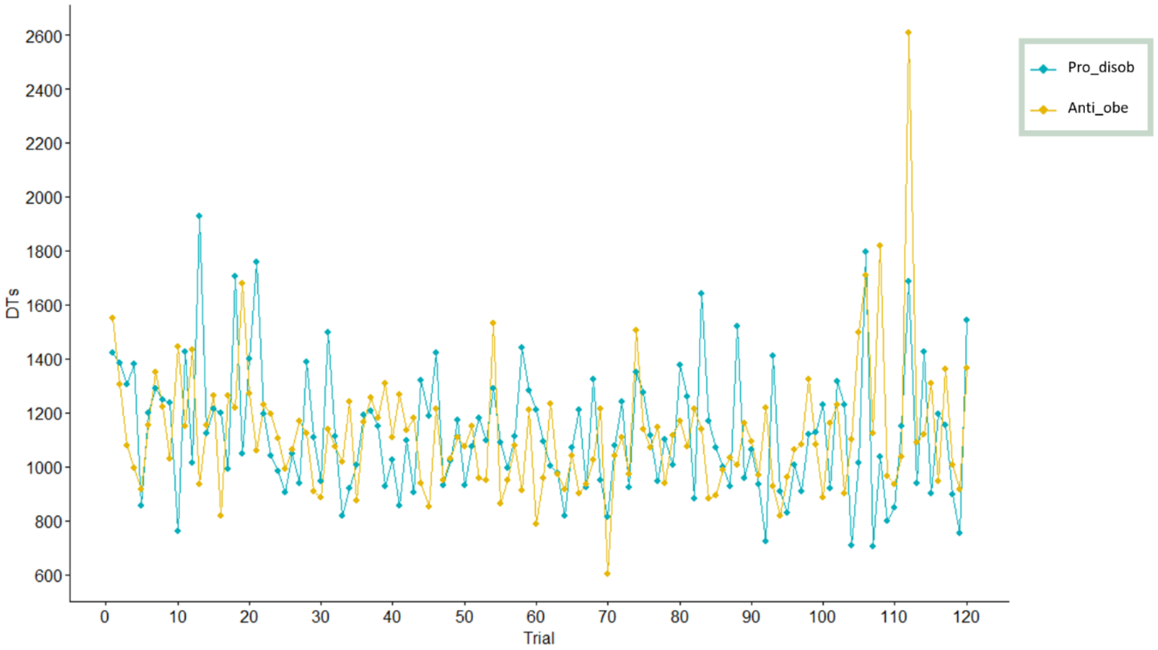

Empathy run

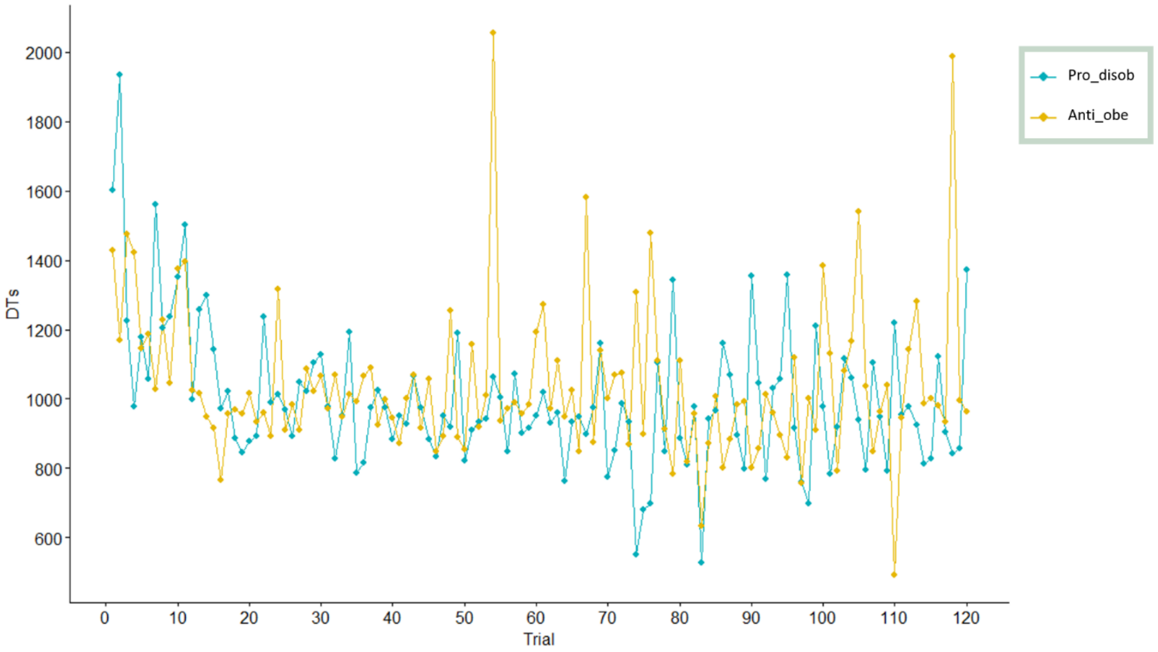

References

Caspar, E. A., Beyer, F., Cleeremans, A., & Haggard, P. (2021). The obedient mind and the volitional brain: {A} neural basis for preserved sense of agency and sense of responsibility under coercion. *PLOS ONE*, *16*(10), e0258884. https://doi.org/10.1371/journal.pone.0258884

Caspar, E. A., Gishoma, D., & de Saldanha da Gama, P. A. (2022). On the cognitive mechanisms supporting prosocial disobedience in a post-genocidal context. *Scientific Reports*, *12*(1), 21875. https://doi.org/10.1038/s41598-022-26460-z

Caspar, E. A., Ioumpa, K., Keysers, C., & Gazzola, V. (2020). Obeying orders reduces vicarious brain activation towards victims' pain. *NeuroImage*, *222*(March). https://doi.org/10.1016/j.neuroimage.2020.117251

Ratcliff, R., & McKoon, G. (2008). The Diffusion Decision Model: Theory and Data for Two-Choice Decision Tasks. *Neural Computation*. https://doi.org/10.1162/neco.2008.12-06-420

Ratcliff, R., & Rouder, J. N. (1998). Modeling Response Times for Two-Choice Decisions. *Psychological Science*, *9*(5), 347–356. https://doi.org/10.1111/1467-9280.00067

Smith, P. L., Ratcliff, R., & Wolfgang, B. J. (2004). Attention orienting and the time course of perceptual decisions: Response time distributions with masked and unmasked displays. *Vision Research*, *44*(12), 1297–1320. https://doi.org/10.1016/j.visres.2004.01.002

Thomson, D. R., Besner, D., & Smilek, D. (2015). A Resource-Control Account of Sustained Attention: Evidence From Mind-Wandering and Vigilance Paradigms. *Perspectives on Psychological Science*, *10*(1), 82–96. https://doi.org/10.1177/1745691614556681

Yamashita, A., Rothlein, D., Kucyi, A., Valera, E. M., Germine, L., Wilmer, J., DeGutis, J., & Esterman, M. (2021). Variable rather than extreme slow reaction times distinguish brain states during sustained attention. *Scientific Reports*, *11*(1), 1–13. https://doi.org/10.1038/s41598-021-94161-0
